# Weber number and the outcome of binary collisions between quantum droplets

**DOI:** 10.1038/s41598-022-22904-8

**Published:** 2022-11-02

**Authors:** J. E. Alba-Arroyo, S. F. Caballero-Benitez, R. Jáuregui

**Affiliations:** Departamento de Física Cuántica y Fotónica, Instituto de Física, Universidad Nacional Autónoma de México, 04510 Mexico City, Mexico

**Keywords:** Matter waves and particle beams, Theoretical physics, Ultracold gases

## Abstract

A theoretical analysis of binary collisions of quantum droplets under feasible experimental conditions is reported. Droplets formed from degenerate dilute Bose gases made up from binary mixtures of ultracold atoms are considered. Reliable expressions for the surface tension of the droplets are introduced based on a study of low energy excitations of their ground state within the random phase approximation. Their relevance is evaluated considering an estimation of the expected excitation energy having in mind the Thouless variational theorem. The surface tension expressions allow calculating the Weber number of the droplets involved in the collisions. Several regimes on the outcomes of the binary frontal collisions that range from the coalescence of the quantum droplets to their disintegration into smaller droplets are identified. Atoms losses of the droplets derived from self-evaporation and three-body scattering are quantified for both homo- and hetero-nuclear mixtures. Their control is mandatory for the observation of some interesting effects arising from droplets collisions.

## Introduction

In most circumstances, degenerate states of dilute atomic gases are realized using samples trapped by different means. The question of whether they can also exist in isolation, without a trapping potential, was theoretically answered in the affirmative within two scenarios. The first one corresponds to a homogeneous atomic gas where the two-body scattering length is negative and much larger than the effective two-body interaction radius; then the contribution to the ground state energy due to the three-body correlations could become dominant and provide a self-trapping mechanism^1^. The second scenario corresponds to a condensed Bose–Bose mixture where the interspecies attraction becomes stronger than the geometrical average of the intra-species repulsions^2^. The stabilization mechanism is then provided by quantum fluctuations that are a direct manifestation of beyond mean-field effects^3^. The implementation of the latter mechanism was the basis for the experimental observation of self-bound droplets of ultra cold atoms in homo-nuclear^4,5^ and hetero-nuclear^6^ mixtures of alkali atoms, and in dipolar condensates^7–10^ that required driving the Bose-Einstein condensate (BEC) into the strongly interacting regime.

Collisions of quantum droplets is the only mechanism available in current experiments that allows the direct observation of surface excitations. Up to now, this mechanism has been used to study the crossover between compressible and incompressible regimes^11^. It is expected that in the incompressible regime surface waves occur and surface tension should be a relevant parameter to understand the conditions required for different results of quantum droplet collisions.

Following the ideas implemented by the nuclear physics community to study the expectations of the nuclear liquid drop model^12–14^, in this work we introduce expressions for the low energy surface excitations of a binary mixture of Bose–Bose ultra cold atoms. These expressions are taken as variational *ansatz* that include effects directly due to the thickness of the surface of the droplet and its symmetries.

Since we are interested in excitations from the ground state of the droplet, the variational *ansatz* depends on that function. We evaluate numerically the ground state for a finite number of atoms, ranging from $10^4$–$10^7$ within the extended Gross–Pitaevskii equation (EGPE) that incorporates the Lee–Huang–Yang term (LHY)^3^ in three dimensions. The analysis is performed for binary mixtures of homo- and hetero-nuclear atomic Bose gases that give rise to quantum droplets. In the case of homo-nuclear BEC mixtures, we also obtain the ground state within an effective formalism that considers finite-range effects as developed in Refs^15,16^. General features of the resulting ground states are then evaluated both numerically and using analytical expressions built from numerical ground state functions, since the corresponding densities support a fit by a Boltzmann profile. These expressions allow a direct calculation of the energy of the surface excitations derived from the different *ansatz*. The resulting energies, besides being compared to previous phenomenological expectations, permit both the selection of the most adequate *ansatz* to describe the excitations according to Thouless variational theorem, and the evaluation of the corresponding surface tension.

Atom losses due to self-evaporation—directly dependent on the excitation modes- and three-body recombination effects are analyzed within the EGPE scheme. The time evolution of such losses for experimental relevant conditions is evaluated (Supplementary Material). Finally, the outcomes of frontal binary collisions are explored. Similarly to previous experimental and theoretical studies^11,18^, we find out several regimes for binary front collisions that range from the coalescence of the quantum droplets to their disintegration into smaller droplets. For classical liquid droplets their collisions are usually investigated in terms of the impact parameter and of the Weber number. The latter is a measure of the relative importance of the inertia of a fluid in terms of the kinetic energy compared to its surface tension^19^. In this work, collisions are studied in terms of a generalization of the Weber number $\mathrm {We}$ to the quantum regime. A direct correlation between $\mathrm {We}$ and the collision outcomes is found both for homo- and hetero-nuclear Bose mixtures and in the incompressible and compressible regimes.

## A variational approach to low energy surface excitations of Bose–Bose mixtures in the quantum regime

We are interested in obtaining expressions for the surface tension of a quantum droplet. To that end we shall follow the formalism developed by Berstch in the study of capillary waves in a superfluid^12^, and which was later extended to Fermi systems with spherical symmetry^13^ having in mind the liquid drop model of nuclei. This formalism is based on the random phase approximation (RPA) and the Thouless variational theorem^20^. RPA provides a strategy to decouple the collective motion of a many-body system from their individual motion^21^. In our case, RPA is equivalent to the linearization of the time-dependent equations that define the dynamics of the system in the vicinity of a variational minimum. A contemporary view of the theoretical basis that supports the Thouless theorem and its relevance for many-body theory is revisited in Ref.^22^. To study quantum droplets arising from vacuum fluctuations in a binary mixture of Bose gases, it is useful to implement Thouless variational principle to such a mixture.

### Thouless variational theorem for a binary mixture of Bose gases

In an analysis of the collective modes of nuclei in RPA, Thouless^20^ showed that it is always possible to find a solution of the independent particle model that makes these collective modes as stable, i.e., they have real eigenfrequencies. This occurs because the solution that gives the lowest expectation value of the Hamiltonian also stabilizes the collective modes in RPA. The stability condition allows the construction of the variational principle and assures that the eigenvectors form a complete set in most cases; this assertion requires the proper definition of a scalar product. Thouless variational theorem guarantees that actual excitation energies are a lower bound to the excitation energies that result from using, as an *ansatz*, known expressions of excitations with harmonic time dependence and satisfying adequate boundary conditions.

Consider a binary mixture of Bose gases composed by $N^{(a)}$ and $N^{(b)}$ atoms of each species. Let us assume that the state of the system is approximately described by the Hartree many-body wavefunction

1 $$ \Psi _{N} (\vec{r}_{1} , \ldots ,{\vec{r}}_{{N^{{(a)}} }} ;{\vec{r}}_{1}^{\prime } , \ldots ,\vec{r}_{{N^{{(b)}} }}^{\prime } ) = \left[ {\prod\limits_{{i = 1}}^{{N^{{(a)}} }} {\chi ^{{(a)}} } (\vec{r}_{i} )} \right]\left[ {\prod\limits_{{i = 1}}^{{N^{{(b)}} }} {\chi ^{{(b)}} } (\vec{r}_{i}^{\prime } )} \right].{\text{ }} $$

As order parameters we consider

2 $$\psi ^{(\alpha )}(\vec{r},t) = \sqrt{N^{(\alpha )}} \chi ^{(\alpha )}(\vec{r},t),\quad \quad \alpha = a, b,$$

$\psi ^{(\alpha )}$ evolves according to the equation

3a $$i\hbar \partial _t \psi ^{(\alpha )}(\vec{r},t)= {\hat{H}}_0^\alpha \psi ^{(\alpha )}(\vec{r},t) + \hat{ U}^\alpha \psi ^{(\alpha )}(\vec{r},t) $$

3b $${\hat{H}}_0^\alpha = -\frac{\hbar ^2}{2m_\alpha }\nabla ^2 + V^\alpha _{ext}(\vec{r}),$$

3c $$\hat{ U}^\alpha \psi ^{(\alpha )}(\vec{r},t)= \int dr^{\prime 3} \Big ({\mathcal {U}}_{\alpha \alpha }(\vec{r}, \vec{r}^\prime ;\rho ^{(\alpha )}(\vec{r}^\prime ,t)) + {\mathcal {V}}_{\alpha \beta }(\vec{r}, \vec{r}^\prime ;\rho ^{(\alpha )}(\vec{r}^\prime ,t),\rho ^{(\beta )}(\vec{r}^\prime ,t))\Big )\psi ^{(\alpha )}(\vec{r},t) $$

In Eq. (3a), $V^\alpha _{ext}(\vec{r})$ is the external potential and $\rho ^{(\alpha )}=\psi ^{(\alpha )*}\psi ^{(\alpha )}$ the density of $\alpha$-atoms. The real functionals ${\mathcal {U}}_{\alpha \alpha }$ and ${\mathcal {V}}_{\alpha \beta }$ in the integral operator $\hat{U}^\alpha$ may incorporate, in an effective way, interactions beyond the standard mean field approximation. The functional ${\mathcal {V}}_{\alpha \beta }$ represents the interaction between different species, i.e. $\alpha \ne \beta$. In the case of EPGE with LHY, the density dependent interactions are superpositions of contact terms^2^,

4a $$\begin{aligned} {\mathcal {U}}_{\alpha \alpha }&= g_{\alpha \alpha }\rho ^{(\alpha )}(\vec{r}^\prime ) \delta ^3(\vec{r} - \vec{r}^\prime )\end{aligned}$$

4b $$\begin{aligned} {\mathcal {V}}_{\alpha \beta }&= \left[ g_{\alpha \beta }\rho ^{(\beta )}(\vec{r}^\prime ) +\frac{4m_\alpha ^{3/5}g_{\alpha \alpha }}{3\pi ^2\hbar ^3}\left( m_\alpha ^{3/5}g_{\alpha \alpha }\rho ^{(\alpha )}(\vec{r}^\prime )+m_\beta ^{3/5}g_{\beta \beta }\rho ^{(\beta )}(\vec{r}^\prime )\right) ^{3/2}\right] \delta ^3(\vec{r} - \vec{r}^\prime ). \end{aligned}$$

The stationary ground state solutions of Eq. (3) define the chemical potentials $\mu _\alpha$,

5 $$\begin{aligned} \psi ^{(\alpha )}_0 (\vec{r},t)= e^{-i\mu _\alpha t/\hbar }\phi ^{(\alpha )}_0(\vec{r}),\quad \quad \alpha = a, b. \end{aligned}$$

Let us consider collective Bogolubov excitations with a harmonic time dependence,

6 $$\begin{aligned} \psi ^{(\alpha )}_{exc} (\vec{r},t)= e^{-i\mu _\alpha t/\hbar }\left( \phi ^{(\alpha )}_0(\vec{r}) + \sqrt{N^{(\alpha )}} \left( u_q^{(\alpha )}(\vec{r}) e^{-i\omega _q t} + v_q^{(\alpha )*}(\vec{r}) e^{i\omega _q t}\right) \right) . \end{aligned}$$

In this equation *q* is a label that determine the characteristics of the excitation derived, e. g., from its geometry. The excitation functions belong to the space expanded by a normalized and complete basis set $\{u_q^a,v_q^a,u_q^b,u_q^b\}$, according to the scalar product

7 $$\begin{aligned} \int d^3r \left[ u_q^{(\alpha )}(\vec{r})u_p^{(\alpha ) *}(\vec{r}) -v_q^{(\alpha )}(\vec{r})v_p^{(\alpha ) *}(\vec{r})\right] = \frac{\omega _q}{\vert \omega _q\vert } \delta _{qp}, \quad \omega _q\ne 0. \end{aligned}$$

Excitations with $\omega _q = 0$ are assumed to be orthogonal to all other excitations with a different set of quantum numbers.

If $\phi ^{(\alpha )}_0$ is a real function, the linear response approximation around the stationary function $\psi ^{(\alpha )}_0 (\vec{r},t)$ is equivalent to the RPA approximation. Taking into account the real character of functionals ${\mathcal {U}}_{\alpha \alpha }$ and ${\mathcal {V}}_{\alpha \beta }$, and factorizing the terms that are either proportional to $e^{ i\omega _q t}$ or $e^{-i\omega _q t}$, Eq. (3) in the RPA approximation is equivalent to the equations

8a $$\begin{aligned}&\hbar \omega _q \zeta _q^{(\alpha )}= {\hat{\Delta }}_0^\alpha \eta _q^{(\alpha )} +2 \phi ^{(\alpha )}_0\int d^3r^\prime \left[ \left[ \frac{\delta {\mathcal {U}}_{\alpha \alpha }}{\delta \rho ^{(\alpha )}} + \frac{\delta {\mathcal {V}}_{\alpha \beta }}{\delta \rho ^{(\alpha )}}\right] \delta \rho ^{(\alpha )}_{q;eff}(\vec{r}^\prime ) \right] +2\phi ^{(\alpha )}_0\int d^3r^\prime \left[ \frac{\delta {\mathcal {V}}_{\alpha \beta }}{\delta \rho ^{(\beta )}}\delta \rho ^{(\beta )}_{q;eff}(\vec{r}^\prime ) \right] ,\end{aligned}$$

8b $$\begin{aligned}&\hbar \omega _q \eta _q^{(\alpha )}={\hat{\Delta }}_0^\alpha \zeta _q^{(\alpha )} ,\end{aligned}$$

8c $$\begin{aligned} \eta _q^{(\alpha )}&= u_q^{(\alpha )} + v_q^{(\alpha )},\,\, \zeta _q^{(\alpha )}=u_q^{(\alpha )}-v_q^{(\alpha )}\end{aligned}$$

8d $$\begin{aligned}&{\hat{\Delta }}_0^\alpha = {\hat{H}}_0^\alpha + \int dr^{\prime 3}\big ({\mathcal {U}}_{\alpha \alpha }(\vec{r}, \vec{r}^\prime ;\rho ^{(\alpha )}_0(\vec{r}^\prime )) + {\mathcal {V}}_{\alpha \beta }(\vec{r}, \vec{r}^\prime ;\rho ^{(\alpha )}_0(\vec{r}^\prime ),\rho ^{(\beta )}_0(\vec{r}^\prime )\big ) - \mu _\alpha , \end{aligned}$$

$\delta \rho ^{(\alpha )}_{q;eff}= \phi ^{(\alpha )}_0\eta _q^{(\alpha )}$ could be directly interpreted as an the effective variation of the density for dynamical purposes since it multiplies the variational derivatives of ${\mathcal {U}}_{\alpha \alpha }$ and ${\mathcal {V}}_{\alpha \beta }$. In fact, using ${\hat{\Delta }}_0^\alpha \phi ^{(\alpha )}_0=0$, the continuity equation

9a $$\begin{aligned}&\frac{\partial }{\partial t}\left[ \delta \rho ^{(\alpha )}_{q;eff}e^{-i\omega _q t}\right] +\vec{\nabla }\cdot \vec{J}_{q;eff}^{(\alpha )} =0, \end{aligned}$$

9b $$\begin{aligned}&\vec{J}_{q;eff}^{(\alpha )} =\frac{1}{2m_\alpha }\left[ \eta _q^{(\alpha )} \left( -i\hbar \vec{\nabla }\right) \phi ^{(\alpha )}_0 -\phi ^{(\alpha )}_0\left( -i\hbar \vec{\nabla }\right) \eta _q^{(\alpha )}\right] , \end{aligned}$$

is derived; it implies a conservation relation for both the real and imaginary parts of $\int d^3x \delta \rho ^{(\alpha )}_{q;eff}e^{-i\omega _q t}$.

Thouless variational theorem^20^ is now applied. In the system under consideration, the application of this theorem is based on the stability inherited to $\psi ^{(\alpha )}_0 (\vec{r},t)$ from the stationary character of the ground state solutions of Eq. (3). The energy $\hbar \omega _q$ is estimated after from Eq. (8) solving for $\omega _q$, and averaging the estimates obtained from each atoms species, $\alpha = a,b$. The variational condition is then written as

10a $$\begin{aligned}&\hbar \omega _{exact}\le \frac{1}{2} \frac{\sum _{\alpha ,\beta =a,b} \langle \eta _q^{(\beta )}\vert {\hat{\Xi }}^{\alpha \beta }\vert \eta _q^{(\alpha )}\rangle + \sum _{\alpha = a,b}\langle \zeta _q^{(\alpha )}\vert {\hat{\Delta }}_0^\alpha \vert \zeta _q^{(\alpha )}\rangle }{\sum _{\alpha =a,b} \langle \zeta _q^{(\alpha )}\vert \eta _q^{(\alpha )}\rangle } =:\hbar \omega _{\eta \zeta }\end{aligned}$$

10b $$\begin{aligned} \langle \eta _q^{(\beta )}\vert {\hat{\Xi }}^{\alpha \beta }\vert\eta _q^{(\alpha )}\rangle &= \delta _{\alpha \beta } \langle \eta _q^{(\beta )}\vert {\hat{\Delta }}_0^\alpha \vert \eta _q^{(\alpha )}\rangle + 2 \int d^3r \eta _q^{(\beta )*}(\vec{r}) \phi _0^{(\alpha )}(\vec{r}) \int d^3r^\prime \left( \frac{\delta {\mathcal {U}}_{\alpha \alpha }}{\delta \rho ^{(\alpha )}} +\frac{\delta {\mathcal {V}}_{\alpha \beta }}{\delta \rho ^{(\alpha )}}\right) \eta _q^{(\alpha )}(\vec{r}^\prime ) \phi _0^{(\alpha )}(\vec{r}^{\prime }) \Big ] \nonumber \\&\quad +2 \int d^3r \eta _q^{(\beta )*}(\vec{r}) \phi _0^{(\alpha )}(\vec{r}) \int d^3r^\prime \left[ \frac{\delta {\mathcal {V}}_{\alpha \beta }}{\delta \rho ^{(\beta )}} \eta _q^{(\beta )}(\vec{r}^{\prime }) \phi _0^{(\beta )}(\vec{r}^{\prime })\right] . \end{aligned}$$

The exact expressions for $u_q$ and $v_q$ would satisfy Eqs. (8) and they are unknown. In a variational calculation, an *ansatz* for the variational functions $\eta _q^{(\alpha )}$ and $\zeta _q^{(\alpha )}$, Eq. (8c), is done incorporating in as much as possible the expected physical characteristics of the particular system under consideration. Our *ansatz* for the excitation functions depends functionally on $\phi _0$ and its derivatives and takes as variational parameters a relative weight between $\eta _q^{(\alpha )}$ and $\zeta _q^{(\alpha )}$ for each atomic species $\alpha =a,b$,

11 $$\begin{aligned} \eta _q^{(\alpha )} = \eta _q^{(\alpha )}\left[ \phi _0^{(\alpha )},\partial \phi _0^{(\alpha )}\right] ,\quad \zeta _q^{(\alpha )} = \gamma _\alpha {\tilde{\zeta }}_q^{(\alpha )}\left[ \phi _0^{(\alpha )},\partial \phi _0^{(\alpha )}\right] . \end{aligned}$$

By requiring that $\gamma _\alpha$ yields a minimum for $\omega _{\eta \zeta }$ it results

12 $$ \gamma _a=  C_a\sqrt{\frac{AB_b}{B_a\left( B_b C_a^2 + B_aC_b^2\right) }},\quad \gamma _b =\frac{B_aC_b}{B_bC_a}\gamma _a, $$

13 $$A=  \sum _{\alpha ,\beta =a,b} \left\langle \eta _q^{(\alpha )}\vert {\hat{\Xi }}^{\alpha \beta }\vert \eta _q^{(\beta )}\right\rangle ,\quad B_\alpha = \left\langle {\tilde{\zeta }}^{(\alpha )}_q\vert {\hat{\Delta }}_0^\alpha \vert {\tilde{\zeta }}^{(\alpha )}_q\right\rangle ,\quad C_\alpha =\left\langle {\tilde{\zeta }}_q^{(\alpha )}\vert \eta _q^{(\alpha )}\right\rangle . $$

and the corresponding optimal variational excitation energy is then

14 $$\begin{aligned} \hbar ^2\omega _{opt}^2 = \frac{AB_aB_b}{B_aC_b^2 + B_bC_a^2}. \end{aligned}$$

### A pair of variational ansatz for surface excitations

Since quantum droplets exhibit different regimes, e. g. compressible and incompressible, we decided to explore the relevance of two pairs of excitation functionals $\eta ^{(\alpha )}_q$ and ${\tilde{\zeta }}^{(\alpha )}_q$, both of them with the simple structure,

15 $$\begin{aligned} \eta ^{(\alpha )}_q = \vec{F}_q^\alpha (\vec{r})\cdot \xi ^{(\alpha )}\vec{\nabla }\phi _0^{(\alpha )}, \quad {\tilde{\zeta }}^{(\alpha )}_q = \Lambda ^\alpha _q(\vec{r})\phi _0^{(\alpha )}. \end{aligned}$$

The functions $\vec{F}^\alpha (\vec{r})$ and $\Lambda ^\alpha (\vec{r})$ incorporate geometric properties of the quantum fluid. The parameter $\xi ^{(\alpha )}$ corresponds to a characteristic scale length for the $\alpha$-species. For these expressions for $\eta ^{(\alpha )}_q$ and $\zeta ^{(\alpha )}_q=\gamma _\alpha {\tilde{\zeta }}^{(\alpha )}_q$,

16 $$\begin{aligned} \delta \rho _{q;eff}^{(\alpha )} = \frac{\xi ^{(\alpha )}}{2} \vec{F}_q^\alpha \cdot \vec{\nabla }\left| \phi _0^{(\alpha )}\right| ^2, \quad \vec{J}_{q;eff}^{(\alpha )} =\left| \phi _0^{(\alpha )}\right| ^2\left[ \frac{1}{2} \gamma _\alpha \left( i\frac{\hbar }{m_\alpha }\vec{\nabla }\right) \Lambda ^\alpha _q\right] =:\vert \phi _0^{(\alpha )}\vert ^2 \vec{v}^{(\alpha )}. \end{aligned}$$

Thus, each component of $\vec{F}_q^\alpha$ would determine the variations of the density $\vert \phi _0^{(\alpha )}\vert ^2$ along different spatial directions, and the “velocity” field $\vec{v}^{(\alpha )}(\vec{r})$ is determined by the gradient of $\Lambda _q^\alpha$, i.e. $\vec{v}^{(\alpha )}(\vec{r})\propto \vec{\nabla }\Lambda _q^\alpha$.

If a sphere delimits the boundary of the $\alpha$-fluid there are two alternative configurations. In the case the fluid is contained within the sphere (droplet) or outside it (bubble). For a droplet $\Lambda ^\alpha _q$ is taken as the solution of Laplace equation in spherical coordinates that is regular at the origin

17 $$\begin{aligned} \Lambda _{\ell m}^\alpha (\vec{r}) = {\mathcal {A}}_{\ell m} Y_{\ell m}(\theta ,\varphi ) \left( \frac{r}{\xi ^{(\alpha )}}\right) ^\ell , \end{aligned}$$

with ${\mathcal {A}}_{\ell m}$ a normalization constant to be evaluated according to Eq. (7). Even though in the present study we focus on quantum droplets, the bubble configuration could result useful for the description of the mixed-bubble regime in which bubbles of the mixed phase coexist with a pure phase of one of the components^23^. In such a case, one would take $\Lambda _{\ell m}^\alpha (\vec{r}) ={\mathcal {A}}_{\ell m} Y_{\ell m}(\theta ,\varphi ) (r/\xi ^{(\alpha )})^{-(\ell +1)}$.

Concerning the function $\vec{F}^\alpha _{\ell m}(\vec{r})$, two reasonable *ansatz* for spherical boundaries are identified,

**Ansatz 1**.

18 $$\begin{aligned} \vec{F}^\alpha _{\ell m}(\vec{r}) = {\mathcal {A}}^{(1)}_{\ell m} Y_{\ell m}(\theta ,\varphi ){\hat{r}} \Rightarrow \eta ^{(\alpha )}_{\ell m} ={\mathcal {A}}^{(1)}_{\ell m} Y_{\ell m}\xi ^{ (\alpha )}\partial _r \phi ^{ (\alpha )}_0\; \text {and}\;\zeta ^{(\alpha )}_{\ell m}=\gamma _\alpha {\mathcal {A}}^{(1)}_{\ell m} Y_{\ell m}(r/\xi ^{ (\alpha )})^\ell \phi ^{ (\alpha )}_0. \end{aligned}$$

It is equivalent to an initially infinitesimal movement of the droplet surface in the radial direction with an amplitude proportional to the angular solution of the wave equation, i.e., a spherical harmonic. This *ansatz* resembles that discussed by Berstch^12^ for a quantum fluid whose surface is defined by the $z=0$ plane and for which $\eta = \partial _z\phi _0 e^{ikx}$.

**Ansatz 2**.

19 $$\begin{aligned} \vec{F}^\alpha _{\ell m}(\vec{r})= \xi ^{(\alpha )}\vec{\nabla }\Lambda _{\ell m}^\alpha \Rightarrow \eta ^{(\alpha )}_{\ell m} ={\mathcal {A}}^{(2)}_{\ell m} Y_{\ell m}[(\xi ^{ (\alpha )}\partial _r) (r/\xi ^{ (\alpha )})^\ell ] [(\xi ^{ (\alpha )}\partial _r) \phi ^{ (\alpha )}_0]\; \text {and}\;\zeta ^{(\alpha )}_{\ell m}=\gamma _\alpha {\mathcal {A}}^{(2)}_{\ell m} Y_{\ell m}(r/\xi ^{ (\alpha )})^\ell \phi ^{ (\alpha )}_0. \end{aligned}$$

This *ansatz* is equivalent to $\delta \rho _{q;eff}^{(\alpha )} \sim \vec{v}^{(\alpha )}\cdot \nabla \vert \phi _0^{(\alpha )}\vert ^2\sim d\vert \phi _0^{(\alpha )}\vert ^2/dt$, i. e., the surface of the droplet is displaced along the velocity field. Note that the second *ansatz* privileges an hydrodynamic interpretation, Eq. (19), while the first *ansatz* at $t=0$ yields an infinitesimal deformation of the droplet perpendicular to its surface, Eq. (18).

A direct calculation shows that, in case the dynamics is governed by contact interactions as it occurs for the EGPE, the contribution of the interaction terms of these excitation functions to *A* and $B_\alpha$, Eq. (13), is null. However, information about such interaction terms is still contained in the stationary solution $\phi _0^{(\alpha )}$. For quantum droplets that can be generated without an external potential, the ground state order parameters depend only on the radial coordinate and are proportional to each other,

20 $$\begin{aligned} \phi ^{(a)}_0 = \sqrt{\frac{N^{(a)}}{N^{(b)}}}\phi ^{(b)}_0 \end{aligned}$$

the last equality being a consequence of Eq. (2). Under these conditions, the excitation energies become

**Ansatz 1.**

21 $$\begin{aligned} \hbar ^2 \omega ^2 \le - \ell (\ell -1)(\ell +2)\frac{ \hbar ^2}{m_a m_b} \frac{ N^{(a)}m_b+ N^{(b)} m_a }{N^{(a)}m_a + N^{(b)}m_b} \frac{ \int dr (\partial _r \phi ^{(a)}_0)^2 \int dr \partial _r \rho ^{(a)}_0 r^{2\ell +1} }{ \left( \int dr \partial _r \rho ^{(a)}_0 r^{\ell +2} \right) ^2 } =\hbar ^2 \omega ^2_{A1}=\epsilon ^2_{A1} \end{aligned}$$

**Ansatz 2.**

22 $$\begin{aligned} \hbar ^2 \omega ^2 \le -\ell (\ell -1) (2\ell +1) \frac{ \hbar ^2}{m_a m_b} \frac{ N^{(a)}m_b+ N^{(b)} m_a}{N^{(a)}m_a + N^{(b)}m_b} \frac{ \int dr \, (\partial _r \phi ^{(a)}_0 )^2 r^{2\ell -2} }{ \int dr \, \partial _r \rho ^{(a)}_0 r^{2\ell +1} }=\hbar ^2 \omega ^2_{A2}=\epsilon ^2_{A2}, \end{aligned}$$

whenever an equal length scale

23 $$\begin{aligned} \xi ^{(a)} = \xi ^{(b)} =\xi , \end{aligned}$$

is chosen for both species. In these equations we notice the presence of the total mass of the droplet

24 $$\begin{aligned} M_T = N^{(a)}m_a + N^{(b)}m_b. \end{aligned}$$

### Surface tension

In his seminal work^24^, Lord Rayleigh considered a classical spherical droplet with constant density of particles $\rho _0$ with mass *m* and a radius $R_0$. For the frequency of excitation $\omega _{exc}$, he showed that

25 $$\begin{aligned} \omega _{exc}^2 = \ell (\ell -1)(\ell +2)\frac{\sigma }{m\rho _0R_0^3} \end{aligned}$$

and he identified $\sigma$ as the surface tension. Lord Rayleigh obtained such an structure for $\omega _{exc}$ analyzing the lowest energy surface excitations of a classical droplet via a variational theorem. The term

$$\begin{aligned} m\rho _0R_0^3 =\frac{3}{4\pi } M_T \end{aligned}$$

is directly related to the total mass of the droplet $M_T$.

We observe that the dependence on the multipole parameter $\ell$ both for *ansatz* 1 and 2, shows that monopole excitations are not surface excitations, and that the $\ell =1$ contribution is null. This is expected since this term corresponds to a translation of the whole droplet. However for $\ell \ge 2$, the estimation value for $\epsilon =\hbar \omega _{exc}$ of the spherical quantum droplet, and as a consequence of the surface tension, is different for each *ansatz*:

1. For *ansatz* 1, the $\ell$-dependence of the coefficient resembles that of Rayleigh and that of the liquid drop model of nuclei in the limit of $N\rightarrow \infty$ nucleons^25^.
2. For *ansatz* 2, the $\ell$-dependence of the coefficient is similar to that predicted for atomic nuclei by Bertsch^13^ and by Casas and Stringari^14^ using RPA and the density-density Green’s function formalism; in both cases, the structure of the second *ansatz* was taken for the excitation modes.

A variational criteria based on the excitation energy can be applied to select between the two excitation *ansatz*. Once adequate expressions for the order parameters $\phi ^{(a)}_0$ are given, the approximate excitation energies are evaluated using both *ansatz*. The variational criteria establishes that the best approximation to the exact description of those excitations is that which provides the lowest excitation energies.

Following Lord Rayleigh ideas, the corresponding surface tension estimation would be given by the expressions

**Ansatz 1.**

26 $$\begin{aligned} \sigma ^{(A1)}_\ell = -\frac{\hbar ^2}{M} \frac{ \int dr (\partial _r \phi ^{(a)}_0)^2 \int dr \partial _r \rho ^{(a)}_0 r^{2\ell +1} }{ \left( \int dr \partial _r \rho ^{(a)}_0 r^{\ell +2} \right) ^2 } \end{aligned}$$

**Ansatz 2.**

27 $$\begin{aligned} \sigma ^{(A2)}_\ell = -\frac{\hbar ^2}{M} \frac{ \int dr \, (\partial _r \phi ^{(a)}_0 )^2 r^{2\ell -2} }{ \int dr \, \partial _r \rho ^{(a)}_0 r^{2\ell +1} } \end{aligned}$$

with

$$\begin{aligned} M = \frac{4\pi }{3}\frac{m_am_b}{N^{(a)}m_b + N^{(b)}m_a}. \end{aligned}$$

The density of a spherical liquid droplet conformed by *N* atoms is characterized by a radius $R_0$, a skin width *dR* and a saturation density. The generic shape of such a droplet can be approximately described by a Boltzmann function,

28 $$\begin{aligned} \rho _B(R;N) = \frac{{\mathcal {B}}_1}{1 + \mathrm {exp}((R-R_0)/dR)}. \end{aligned}$$

For Bose–Bose mixtures of experimental interest and number of atoms ranging from $10^4$–$10^7$, we have performed numerical calculations for the ground state derived from an EGPE for homo- and hetero-nuclear mixtures, and, for the homo-nuclear case, from the effective formalism that considers finite-range effects^15,16^. The resulting density profiles fits the Boltzmann density with a reliability above 0.998 in all considered cases. The resulting parameters are reported in the Supplementary Material. Using Eq. (28), the surface tension Eqs. (26–27) can be written in terms of the Fermi-Dirac integrals

29 $$\begin{aligned} F_s (z)= \frac{1}{\Gamma (s+1)}\int _0^\infty \frac{dx x^s}{1+e^{x-z}}. \end{aligned}$$

So that, for $\ell > 1$

30 $$ \sigma _\ell ^{(A1)}=  \frac{\hbar ^2}{8M dR^4}\frac{(2\ell +1)!}{((\ell +2)!)^2}\left[ 1-\frac{1}{(1+ e^{R_0/dR})^2}\right] \frac{F_{2\ell }(R_0/dR)}{(F_{\ell +1}(R_0/dR))^2}, $$

31 $$\sigma _\ell ^{(A2)}= \frac{\hbar ^2}{16M dR^4}\frac{1}{\ell (4\ell ^2 -1)} \frac{F_{2\ell -3}(R_0/dR)+F_{2\ell -4}(R_0/dR)}{F_{2\ell }(R_0/dR)}, $$

expressions that correspond to *ansatz* 1 and 2 respectively.

### Weber number for Boltzmann shaped droplets

Consider the collision of quantum droplets that initially were radially spaced at a distance $2d_0$; they are assumed to be kicked off as described by a plane wave wavefunction factor,

32 $$\begin{aligned} \Psi (\vec{r}, t=0)&=  \Psi _1(\vec{r}) + \Psi _2(\vec{r}) \nonumber \\&=  \begin{pmatrix}\psi _{a_1}(\vec{r} + \vec{d}_0)\\ \psi _{b_1}(\vec{r}+ \vec{d}_0)\end{pmatrix} e^{i\vec{k}_0\cdot \vec{r}/2} +\begin{pmatrix}\psi _{a_2}(\vec{r} - \vec{d}_0)\\ \psi _{b_2}(\vec{r}- \vec{d}_0\end{pmatrix} e^{-i\vec{k}_0\cdot \vec{r}/2}. \end{aligned}$$

The mean value

33 $$\begin{aligned} {\mathcal {K}} = -\frac{\hbar ^2}{2}\sum _{i=1,2}\int d^3r \Psi _i^\dagger (\vec{r})\begin{pmatrix} \frac{\nabla ^2}{m_{a_i}} &{} 0\\ 0 &{} \frac{\nabla ^2}{m_{b_i}} \end{pmatrix} \Psi _i(\vec{r})\end{aligned}$$

is taken as a measure of the initial kinetic energy of the droplets. Assuming a non significant overlap between the droplets at $t=0$, it can be decomposed as the sum of the translational kinetic energy of each droplet as a whole ${\mathcal {K}}_{trans}$, and an internal kinetic energy of the atoms within the droplets ${\mathcal {K}}_{int}$. Thus, ${\mathcal {K}}\approx {\mathcal {K}}_{trans} + {\mathcal {K}}_{int}$, with

34 $$\begin{aligned} {\mathcal {K}}_{trans}= \frac{\hbar ^2}{2}\left[ \frac{N^{(a_1)}k_0^2}{4m_{a_1}} + \frac{N^{(b_1)}k_0^2}{4m_{b_1}} + \frac{N^{(a_2)}k_0^2}{4m_{a_2}} + \frac{N^{(b_2)}k_0^2}{4m_{b_2}} \right] ,\quad {\mathcal {K}}_{int} = -\frac{\hbar ^2}{2}\sum _{i=1,2}\sum _{\alpha =a,b}\int d^3r\psi ^*_{\alpha _i}(\vec{r})\frac{\nabla ^2}{m_{\alpha _i}}\psi _{\alpha _i}(\vec{r}). \end{aligned}$$

The dimensionless quantity suitable for characterizing binary collisions of classical incompressible droplets is the Weber number^19^. It is a dimesionless quantity for each one of the droplets, defined as the ratio between the translational kinetic energy ${\mathcal {K}}_{trans}$ before the collision and the surface energy of excitation evaluated in terms of the surface tension of the droplet. This definition can be extended to quantum droplets as

35 $$\begin{aligned} \mathrm {We}_\ell =\frac{{\mathcal {K}}_{trans}}{R_0^2\sigma _\ell } \end{aligned}$$

where $R_0$ is the initial radius of the droplet. For the collision of two identical quantum droplets like those described in this work, this expression becomes

**Ansatz 1**

36 $$\begin{aligned} \mathrm {We}_\ell ^{(A1)} = \frac{8\pi (dR k_0)^2}{3}\left( \frac{dR}{R_0}\right) ^2 \frac{((\ell +2)!)^2}{(2\ell +1)!}\left[ 1-\frac{1}{(1+ e^{R_0/dR})^2}\right] ^{-1} \left[ \frac{(F_{\ell +1}(R_0/dR))^2}{F_{2\ell }(R_0/dR)}\right] , \end{aligned}$$

**Ansatz 2**

37 $$\begin{aligned} \mathrm {We}_\ell ^{(A2)}=\frac{16\pi (dR k_0)^2}{3}\left( \frac{dR}{R_0}\right) ^2 (\ell (4\ell ^2 -1)) \frac{F_{2\ell }(R_0/dR)}{F_{2\ell -3}(R_0/dR)+F_{2\ell -4}(R_0/dR)}. \end{aligned}$$

Notice that the expressions of $\mathrm {We}_\ell$ for the ground state of the quantum droplets given by Eqs. (36–37) make evident the relevance of the relation between the de Broglie wavelength and the skin width of the droplet, $k_0 dR$, as a measure of the momentum that could be transfered to the droplet during the collision. The second relevant parameter is the ratio $R_0/dR$ that determines the deformation of the droplet due to a multipole excitation.

## The ground state of a quantum droplet

The theoretical structure developed in the previous Section will be applied to quantum droplets of homo-nuclear ($^{39}$K) and hetero-nuclear ( $^{41}$K and $^{87}$Rb) mixtures as those predicted by^2,5^. The physical parameters of the illustrative examples in this and the following sections are compatible with experiments reported in Refs^4,6^. We consider a mixture of two ultracold Bose gases with atom masses $m_a$ and $m_b$, densities $n_a$ and $n_b$, scattering lengths $\mathrm {a}_{\alpha \beta }$, $\alpha ,\beta =a,b$, and coupling constants $g_{\alpha \beta }= 2\pi \hbar ^2\mathrm {a}_{\alpha \beta }(1/m_a +1/m_b)$ with $g_{aa}> 0$, $g_{bb} > 0$, and $g_{ab}<0$. The first correction to the mean-field contribution to the ground state energy of a homogeneous weakly repulsive Bose gas is given by the LHY term^3^. This term is negligible in many circumstances. However, there are situations where the LHY and the mean field contributions can be of the same order without leaving the weakly interacting regime, $n\mathrm {a}^3\ll 1$. Whenever $g^2_{ab}\sim g_{aa} g_{bb}$ the EGPE which incorporates the LHY term is^6^

38 $$\begin{aligned} i \hbar \partial _t \Psi _\alpha = \left( - \frac{\hbar ^2}{2m_\alpha } \nabla ^2 + g_{\alpha \alpha } |\Psi _\alpha |^2 + g_{\alpha \beta } |\Psi _\beta |^2+ \frac{4}{3 \pi ^2} \frac{m_\alpha ^{3/5} g_{\alpha \alpha }}{ \hbar ^3} \left( m_\alpha ^{3/5} g_{\alpha \alpha } |\Psi _\alpha |^2 + m_\beta ^{3/5} g_{\beta \beta } |\Psi _\beta |^2 \right) ^{3/2} \right) \Psi _\alpha . \end{aligned}$$

The inter- and intra-species coupling constants $g_{\alpha \beta }$ can be independently controlled. The mean-field energy term, which is proportional to the square of the density $n^2$, that is negative and the LHY contribution, which is proportional to $n^{5/2}$ is positive. Then, the quantum LHY repulsion could neutralize the mean-field attraction and stabilize the system against collapse^2^. A less intuitive, albeit more precise description of the self-trapping mechanism can be obtained via Quantum Monte Carlo simulations^15^, they predict similar general properties for the quantum droplets, though different specific scenarios of their occurrence. The general properties are: generic features of the collective modes^9^, the presence of striped states in confined geometries^27^, and the existence of arrays of phase coherent droplets with transient super solid properties^10,28,29^.

The self-trapping regime requires a minimum number of atoms $N_c^{(\alpha )}$ which can be evaluated by calculating the ground state numerically. There is a transient regime characterized by a set of additional critical numbers $N_{ql}^{(\alpha )}$. For $N_{c}^{(\alpha )}<N^{(\alpha )}<N_{ql}^{(\alpha )}$ the atomic density exhibits a surface with a thickness similar to the radius of the droplet. If the quantum droplet has no collective modes with energy lower than the particle emission threshold self-evaporation can occur^2,30^. For higher *N* values, the ground state corresponds to that expected for a liquid either a classical liquid, a helium nano droplet, or an atomic nuclei within the liquid drop model. Then, the self-trapping quantum state is characterized by a core with a quasi uniform density and a thin free surface. According to EGPE Ref.^2^: (1) A spherical shape with radius $R_0\approx ( 3 N/4\pi n_\alpha ^{(0)}\xi ^3)^{1/3}\xi$, is expected with

$$\begin{aligned} \xi =\hbar \sqrt{\frac{3}{2}\frac{\sqrt{g_{bb}}/m_a + \sqrt{g_{aa}}/m_b}{\vert \delta g\vert \sqrt{g_{aa}}n_a^{(0)}}}, \end{aligned}$$

and $\delta g = g_{ab} + \sqrt{g_{aa}g_{bb}}$; (2) a surface thickness of order $\xi$; (3) a saturation density in the limit of $N^{(\alpha )}\rightarrow \infty$

39 $$\begin{aligned} n_\alpha ^{(0)} = \frac{25\pi }{1024} \frac{1}{(1+ (m_b/m_a)^{3/5} \sqrt{g_{bb}/g_{aa}})^5}\frac{1}{\mathrm {a}_{\alpha \alpha }^3}\frac{\delta g^2}{g_{aa}g_{bb}}. \end{aligned}$$

As a consequence, the well-known shape of a Boltzmann function Eq. (28) is a feasible mathematical model of the spherical density profile of a droplet for a given number of particles with $R_0$ and *dR* the radius and suface thickness and the density at the origin, $\rho _B(0;N) = {{{\mathcal {B}}}}_1/(1 + \mathrm {exp}(-R_0/dR))$. Details for specific homo-nuclear and hetero-nuclear BEC mixtures of experimental interest for a finite number of atoms are given in Section SMA of the Supplementary Material. It is worth to mention that the reliability of the Boltzmann density model was found to be above 0.998 in all numerical simulations.

## Low energy excitation modes of quantum droplets

### Variational multipole modes in configuration space

Within the Bogolubov scheme, the excitation modes of the quantum droplets are determined by the normalized and complete basis set $\{u_q^{(a)},v_q^{(a)},u_q^{(b)},u_q^{(b)}\}$. An ideal collective excitation of the wave function $\psi ^{(\alpha )}_{exc}$ shows a harmonic time dependence, Eq. (6).

**Figure 1:**
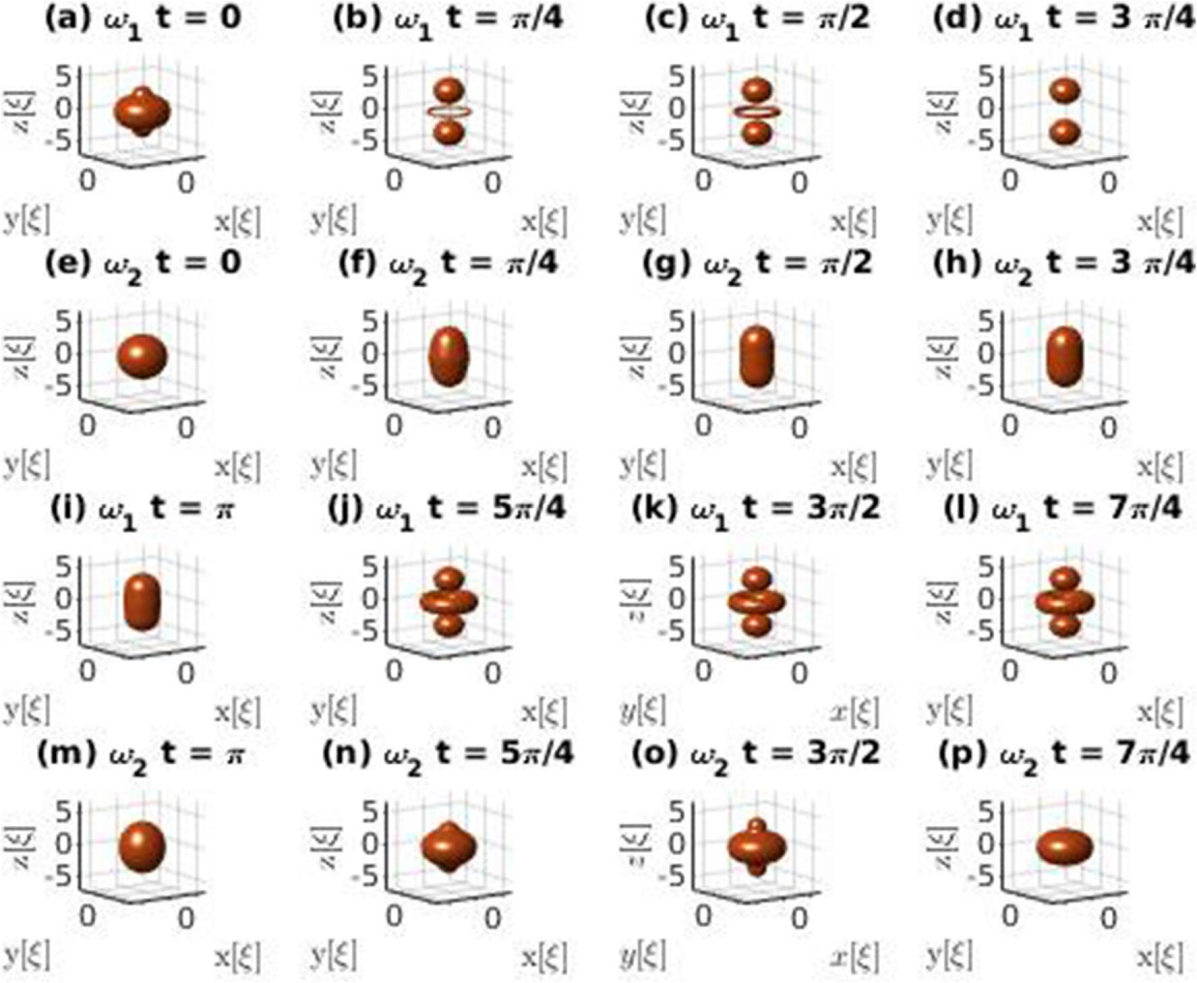
Comparative illustrations of the ideal evolution of the density of a quantum droplet formed by $N^{(Rb)}$ = 96818 and $N^{(K)}$ = 84578 atoms considering the excited quadrupole state, $\ell =2$ and $m_\ell =0$, generated from the ground state droplet. *Ansatz* 1 corresponds to rows (**a**–**d**) and (**i**–**l**) and *ansatz* 2 to rows (**e**–**h**) and (**m**–**p**). In this case $\omega _1 =0.21\tau ^{-1}$ and $\omega _2 =0.22\tau ^{-1}$. The 3D graphs where generated by plotting isodensity surfaces at fifteen percent of the maximum density at each time.

Figure 1 illustrates the resulting ideal evolution of the atomic densities for hetero-nuclear mixtures applied to the ground state wave function $\phi ^{(\alpha )}_0$ of the EGPE being $u_q^{(a)}$ and $v_q^{(a)}$ the functions determined by either Eq. (18) (*ansatz*1:A1) or Eq. (19) (*ansatz*2:A2). The number of atoms in the illustrative case of Fig. 1 corresponds approximately to the minimum required for the ground state droplet to achieve the saturation density. In the Supplementary Material SMB other ideal evolutions for cases of particular relevance in the compressible regime are presented. The unit of time is taken as suggested in Ref.^2^,

40 $$\begin{aligned} \tau =\frac{3\hbar }{2} \frac{\sqrt{g_{aa}} + \sqrt{g_{bb}}}{\vert \delta g\vert \sqrt{g_{aa}}n_a^{(0)}}, \end{aligned}$$

and the unit of energy is $\hbar /\tau$. Graphs are obtained from isodensity surfaces chosen to approximately delimit the droplets. Apparently the droplets seem to divide in several sub-droplets during particular time intervals. However this interpretation is not precise since it is just based in the isodensity surfaces. The more detailed numerical analysis shows that there is always a dilute gas between the apparent fragments. In Fig. 1 we observe clear differences and similarities between the structures of the excitation modes for different *ansatz*.

It is worth mentioning that these ideal evolutions do not take into account atomic losses, which in the experimental realm involve at least two processes: self-evaporation and three-body dispersion. They will be discussed in a following Section and in the Supplementary Material. The case illustrated in Fig. 1 is not expected to show significant self-evaporation. Note, however, that in this study the relevance of ideal droplet evolution under excitation modes is conditional on its ability to illustrate the structure of atomic densities under surface excitations as a result of quantum droplet collisions.

### Excitation energies: variational criteria

**Figure 2:**
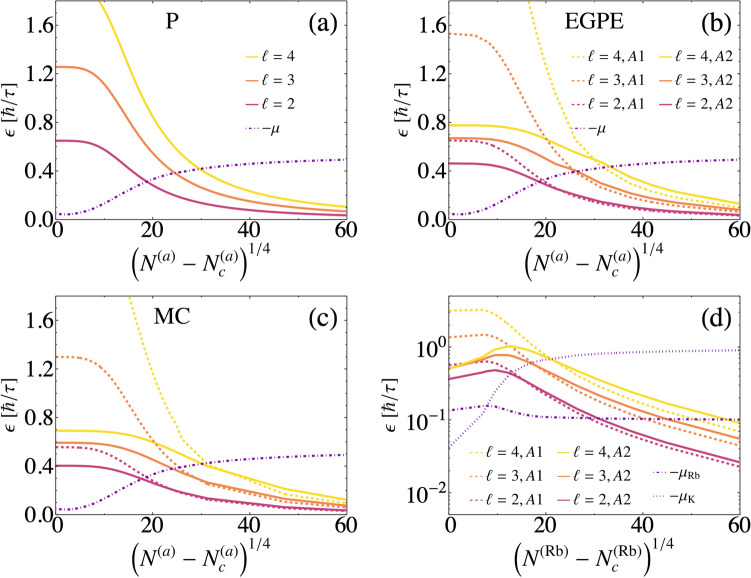
Approximate excitation energies $\epsilon _\ell$ as a function of the number of atoms *N* for (**a**–**c**) a homo–nuclear mixture of $^{39}$K atoms; (**d**) a hetero-nuclear mixture of $^{41}$K and $^{87}$Rb atoms. The negative value of the chemical potential $\mu$ is also shown. $\epsilon _\ell$ is evaluated according to different models: (**a**) a phenomenological expression expected to be valid just for $N\gg N_c$^2^ (P); (**b**, **d**) the variational scheme for the LHY-EGPE, Eqs. (21–22); (**c**) the variational scheme for the effective interaction obtained from Monte-Carlo (MC)^16^. In (**b**–**d**) the results from *ansatz* 1, A1, (dashed) and *ansatz* 2, A2, (continous) are shown. The natural unit $\hbar /\tau$ of energy is used with $\tau =1.308$  ms (homo-nuclear) and $\tau =1.191$ ms (hetero-nuclear).

The ground state wave functions can be used to calculate the energies associated to surface excitations within a description where just contact interactions are considered, i.e., according to *ansatz* 1 (A1), Eq. (21) and *ansatz* 2 (A2), Eq. (22). The numerical results are illustrated in Fig. 2 for the physical parameters given in Section SMA of the Supplementary Material.

For homo-nuclear mixtures, the phenomenological expression

41 $$\begin{aligned} \epsilon _\ell ^{(P)} = \sqrt{\frac{4\pi (1 + \sqrt{3})}{35}\ell (\ell -1)(\ell +2)\frac{n_i^{(0)}\xi ^3}{N}}, \end{aligned}$$

based in RPA is also illustrated. The variational criterium identifies *ansatz* 2 as a better evaluation of the energy for small values of *N*. However, for a given $\ell$ value, there is a critical value $N_\ell ^c$ for which *ansatz* 1 gives a lower excitation energy if $N >N_\ell ^c$. The phenomenological estimation $\epsilon _\ell ^{(P)}$ cannot be considered closer to the exact one even though it may be lower than the result of *ansatz* 1 or 2, both because it is not supported by a variational theorem and because it is expected to be valid just for $N\gg 1$^2,31^; note that the *ansatz* 1 results approximately coincide with $\epsilon _\ell ^{(P)}$ in this limit.

For low *N* calculations where *ansatz* 2 gives lower energies, droplets have a radius $R_0$ similar to its width *dR*, thus the quantum incompressible regime has not been reached. The dependence on $\ell$ of the energy involving the factor $\ell (\ell -1)(\ell +2)$ that was predicted by Lord Rayleigh for classical liquids, is also predicted by *ansatz* 1 and the phenomenological result Eq. (41), but not by *ansatz* 2. As illustrated in Fig. 2, the similarities between the results for the excitation energies obtained for LHY calculations and the effective equation resulting from density Monte Carlo calculations are remarkable. Notice however that there are relevant subtleties. In particular, for given *N* and $\ell$ values the excitation energies of the suggested modes that surpass the absolute value of the chemical potential $\mu$ differ. So that, *N* values for which self-evaporation occurs are similar but not identical to those predicted in Ref.^2^. The differences arise not just from the dynamical equation but also from the natural excitation modes description.

Summarizing, for homo-nuclear mixtures using the variational criteria, both for LHY and Monte Carlo inspired calculations, it results that for $N<N^\ell _c$ ($N>N^\ell _c$) low energy excitations are better described by *ansatz* 2 (*ansatz* 1). We have $N^{\ell =2}_c\sim N_{ql}$ with $N_{ql}$ the critical value between compressible and incompressible regimes.

In the hetero-nuclear case, the behaviour of the two chemical potentials is different for the two species as expected from their differences in mass and coupling interactions. In Fig. 2d, the values of the negative of the chemical potentials $-\mu _K$ and $-\mu _{Rb}$ of the ground states obtained from LHY-EGPE are shown. Notice that $\vert \mu _K\vert$ always increases as $N^{(K)}=\sqrt{g_{RbRb}/g_{KK}} N^{(Rb)}$ increases, while for $^{87}$Rb atoms $\vert \mu _{Rb}\vert$ at $N^{(Rb)}=N_c^{(Rb)}$ is higher than its asymptotic value$~ 0.1\hbar /\tau$ for $N^{(Rb)}\rightarrow \infty$. The crossing point of those chemical potentials corresponds to $N^{(Rb)}-N_c^{(Rb)} \approx 8^4$, that is $N^{(Rb)}\approx 16326\approx 1.3\cdot N_c^{(Rb)}$ which is smaller than the maximum saturation density. That is, for $N_c^{(\alpha )}\le N^{(\alpha )}\le 1.3N_c^{(\alpha )}$ it is energetically favorable to release a single $^{41}$K atom from the fluid, than a $^{87}$Rb atom; the reverse condition applies for higher $N^{(\alpha )}$ values. That is, the differences in the value of the chemical potential indicates that the self-evaporation rates of each species differ and depend on the number of atoms in the droplet. In a similar way than the homo-nuclear case, *ansatz* 2 gives a better estimation of the energy for the compressible regime at small values of the number of atoms, $N^{(Rb)}< 70227$ for quadrupole excitations and $N^{(Rb)}< 10^7$ for octupole excitations. *Ansatz* 1 must be taken as a better variational estimation for high $N^{(a)}$ values. According to the variational criteria, the corresponding Bogolubov functions should be selected to describe the excited state.

In the incompressible regime, the direct numerical analysis of the Bogolubov de Gennes equations Eq. (8) reveals that the energies estimated with the variational criteria reported in this work coincide with the exact ones at the two digit level.

### Atom losses

The experimental observation of the dynamics of quantum droplets before and after a collision is highly constrained by atom losses. Self-evaporation and three-body scattering have been identified as relevant atom losses mechanisms.

Self-evaporation arises whenever the number of atoms in the droplet is such that its excitation energy $\epsilon$ lies in the continuum, i.e., $\epsilon >-\mu$, see Fig. 2. Previously, self-evaporation was studied^26^ using a monopole excitation induced by an isotropic harmonic potential, only radially dependent. Here we benchmark the quadrupole excitation functions introduced in “A pair of variational ansatz for surface excitations” section, and the time evolution within the EGPE scheme of an excited droplet considering angular deformations. The initial state of the atomic cloud was taken as given by Bogolubov expression, Eq. (6) at $t=0$. The evaporation rates are evaluated in terms of the atoms that leave the droplet as a function of time; the latter are identified as those located outside the minimum radius of the sphere that includes all the atoms in the *ideal* evolution of the approximate excitation mode given by the Bogolubov expression. In the Section SMB of the Supplementary Material, the evolution of self-evaporation is illustrated for homo-nuclear and hetero-nuclear mixtures and some interesting processes are highlighted.

For the homo-nuclear case, the biggest atom loss is due to three-body scattering with typical time scale in the 10 ms range. Notice that additional mechanisms that threaten the stability of a droplet arise from changes on the quotient $N^{(a)}/N^{(b)}$ as well as the losing the spatial overlap of the two atomic wave functions that is required for self-trapping. Our numerical simulations confirm that for hetero-nuclear mixtures of $^{87}$Rb and $^{41}$K, there is a time window in the tens of milliseconds scale where the droplet state is preserved, as seen in Section SMC of the Supplementary Material. Therefore, collisions of two droplets could be observed. In fact, experiments of collisions in the compressible regime have already been performed, Ref.^11^.

## Frontal collisions of quantum droplets

The observation of the frontal collision of two quantum droplets were first reported in Ref.^11^. The corresponding experiments involved homo-nuclear mixtures of $^{39}$K atoms in two different hyperfine states. The two droplets were generated from degenerate gases located nearby the minima of a double well potential. Subsequently, the potential barrier was turned off and the two droplets migrate towards the center of a harmonic confining potential. After a controlled time interval $\Delta t$ the potential was turned off, and the droplets collided in a potential free environment; $\Delta t$ was used to tune the initial relative speed of the droplets^32^. The analysis of experimental data in Ref.^11^ in terms of the relative velocity was inspired by a classical Weber number definition, $\mathrm {We} = 2\rho R_0 v^2/\sigma$. It was found that if the speed were higher than a critical value $v_c$ as a result of the collision the droplets coalesced; $v_c$ depends in a non monotonic way on the number of atoms in each initial droplet. This is a consequence of the dependence of $\rho$, $R_0$ and $\sigma$ on *N*, which we have studied in the previous sections. The experiments involved $N\simeq 10^4$ so that the droplets were in the compressible regime; due to large three-body atom losses the whole dynamics was required to involve small time intervals of around 15 ms.

**Figure 3:**
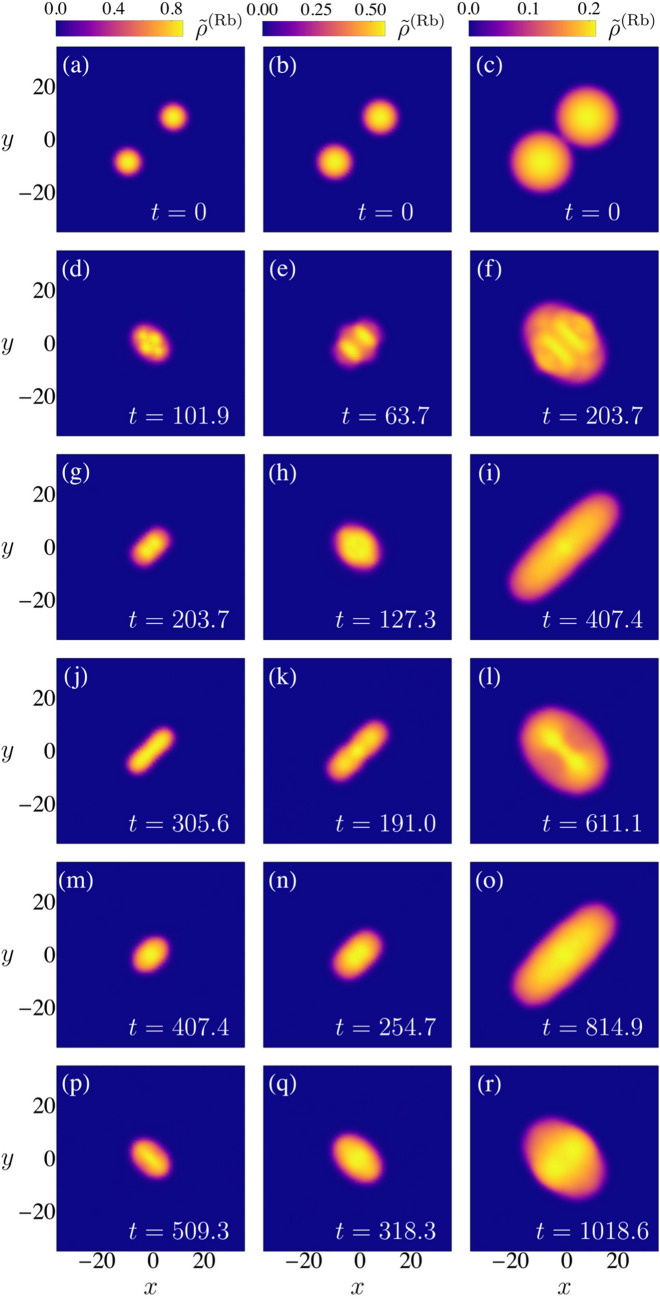
Illustrative examples of frontal collisions of two droplets leading to its coalescence. Identical hetero-nuclear droplets of Rb and K atoms are considered in each column, where the density of Rb atoms integrated along the z-direction ${{\tilde{\rho }}}^{(Rb)}(x,y) = \int dz \rho ^{(Rb)}(x,y,z)$ is depicted at different evolution times. The initial translational kinetic energy is varied to guarantee that for $N^{Rb} = 5 \times 10^5$ (first column), $N^{Rb} = 1 \times 10^6$ (second column) and $N^{Rb} = 5 \times 10^6$ (third column) the Weber number equals $\mathrm {We}_2^{(A1)}= 6.2$.

**Figure 4:**
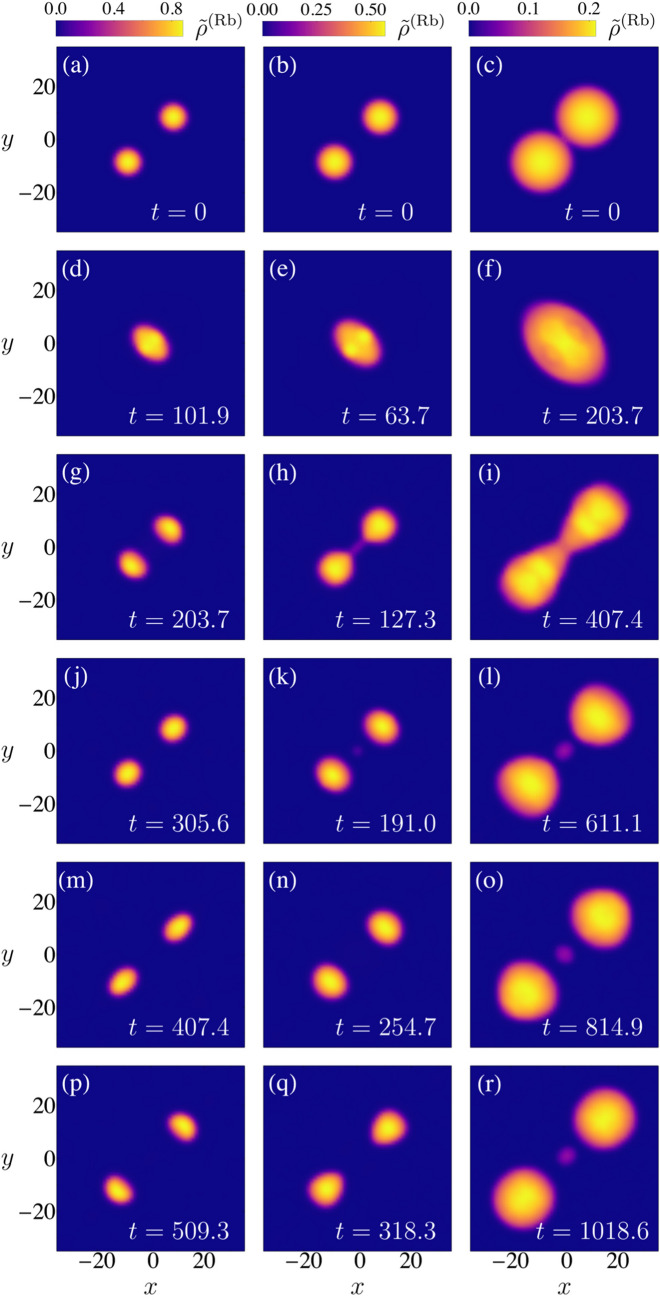
Illustrative examples of frontal collisions of two droplets leading to an initial coalescence followed by a disintegration of the resulting droplet into two droplets that are then expelled in opposite directions. Initially, identical hetero-nuclear droplets of Rb and K atoms are considered in each column, where the density of Rb atoms integrated along the z-direction ${\tilde{\rho }}^{(Rb)} (x, y) = \int dz \rho ^{(Rb)} (x, y, z)$ is depicted at different evolution times. The initial translational kinetic energy is varied to guarantee that for $N^{(Rb)} = 5 \times 10^5$ (first column), $N^{(Rb)} = 1 \times 10^6$ (second column) and $N^{(Rb)} = 5 \times 10^6$ (third column), the Weber number equals $\hbox {We}^{(A1)}_2 =14.5$.

**Figure 5:**
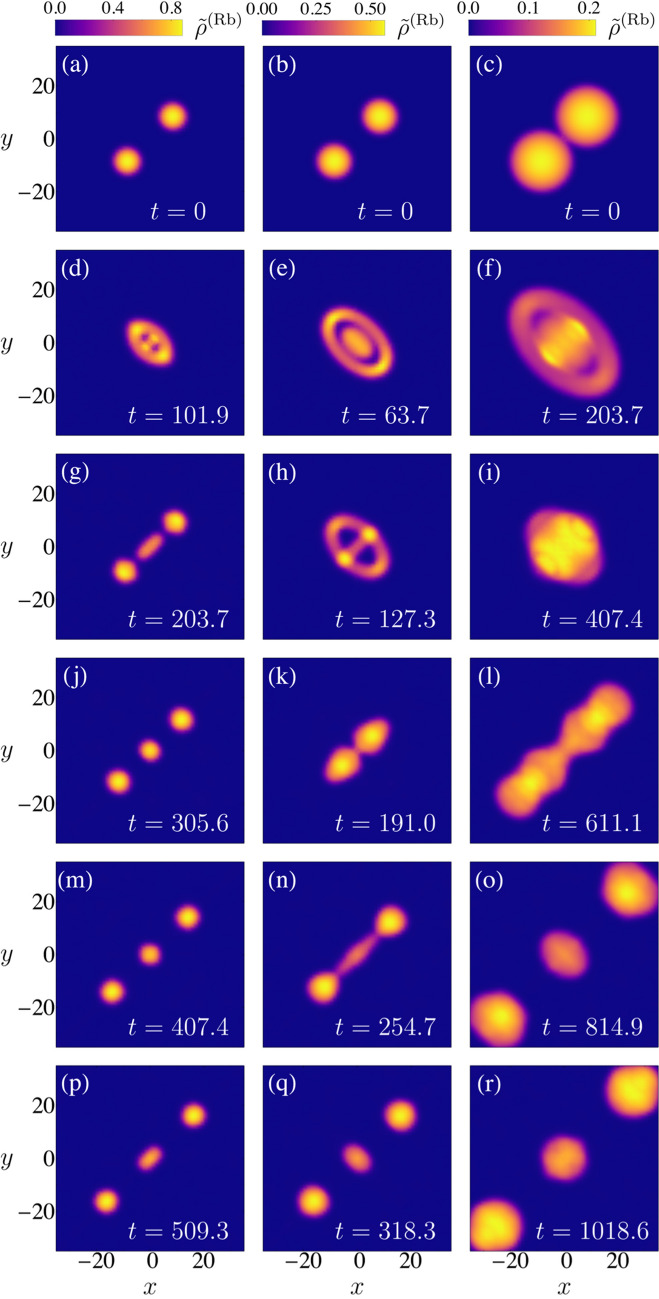
Illustrative examples of frontal collisions of two droplets leading to an initial coalescence followed by a disintegration of the resulting droplet into two droplets that are then expelled in opposite directions and a third droplet is formed at the center.. Initially, identical hetero-nuclear droplets of Rb and K atoms are considered in each column, where the density of Rb atoms integrated along the z-direction ${{\tilde{\rho }}}^{(Rb)}(x,y) = \int dz \rho ^{(Rb)}(x,y,z)$ is depicted at different evolution times. The initial translational kinetic energy is varied to guarantee that for $N^{Rb} = 5 \times 10^5$ (first column), $N^{Rb} = 1 \times 10^6$ (second column) and $N^{Rb} = 5 \times 10^6$ the Weber number equals $\mathrm {We}_2^{(A1)}= 31.9$.

### Frontal collisions of two droplets with and without out atom losses by three-body scattering

At first, we have studied the effects of binary collisions of quantum droplets in an EGPE scheme described by Eqs. (38) without including three body atom losses. We performed simulations for homo-nuclear ($^{39}$K) and hetero-nuclear ($^{41}$K–$^{87}$Rb) systems, with the parameters reported in Section SMA of the Supplementary Material. Different initial kinetic energies and number of particles were studied always in the incompressible regime where no self-evaporation is expected. The initial condition corresponds to identical ground state droplets with an overall movement described by Eq. (32). Later on, simulations including the experimental three-body scattering coefficients $K_{\alpha \beta \gamma } \ne 0$ were performed.

In general, for the kinetic energies here considered, the simulations yield different possible final results of a frontal collision,

1. Coalescence: Formation of a single droplet from two colliding droplets. The resulting droplet is found to be created mainly in a quadrupole excitation, although higher order modes could also be present (see Fig. 3 ).
2. Disintegration into two droplets after coalescence: after the collision, the oscillations of the resultant coalesced droplet are so strong that it breaks into two similar droplets. These two droplets exit in opposite directions and leave the collision zone with a translational movement described mainly as a dipole like oscillation (See Fig. 4).
3. Disintegration into three droplets after coalescence: when the initial droplets are large and the collision is energetic enough, after the breaking and the expulsion of two droplets in opposite directions, a third droplet is formed at the center. The latter oscillates mainly in a quadrupole mode as in the first case (illustrated in Fig. 5). Disintegration into more droplets is expected for even larger translational kinetic energies.

Similar results for homo-nuclear droplets are described in Refs^11,18^. In the simulations no quasi-elastic collisions were observed, though they have been predicted to occur in one dimensional simulations^34^.

**Figure 6:**
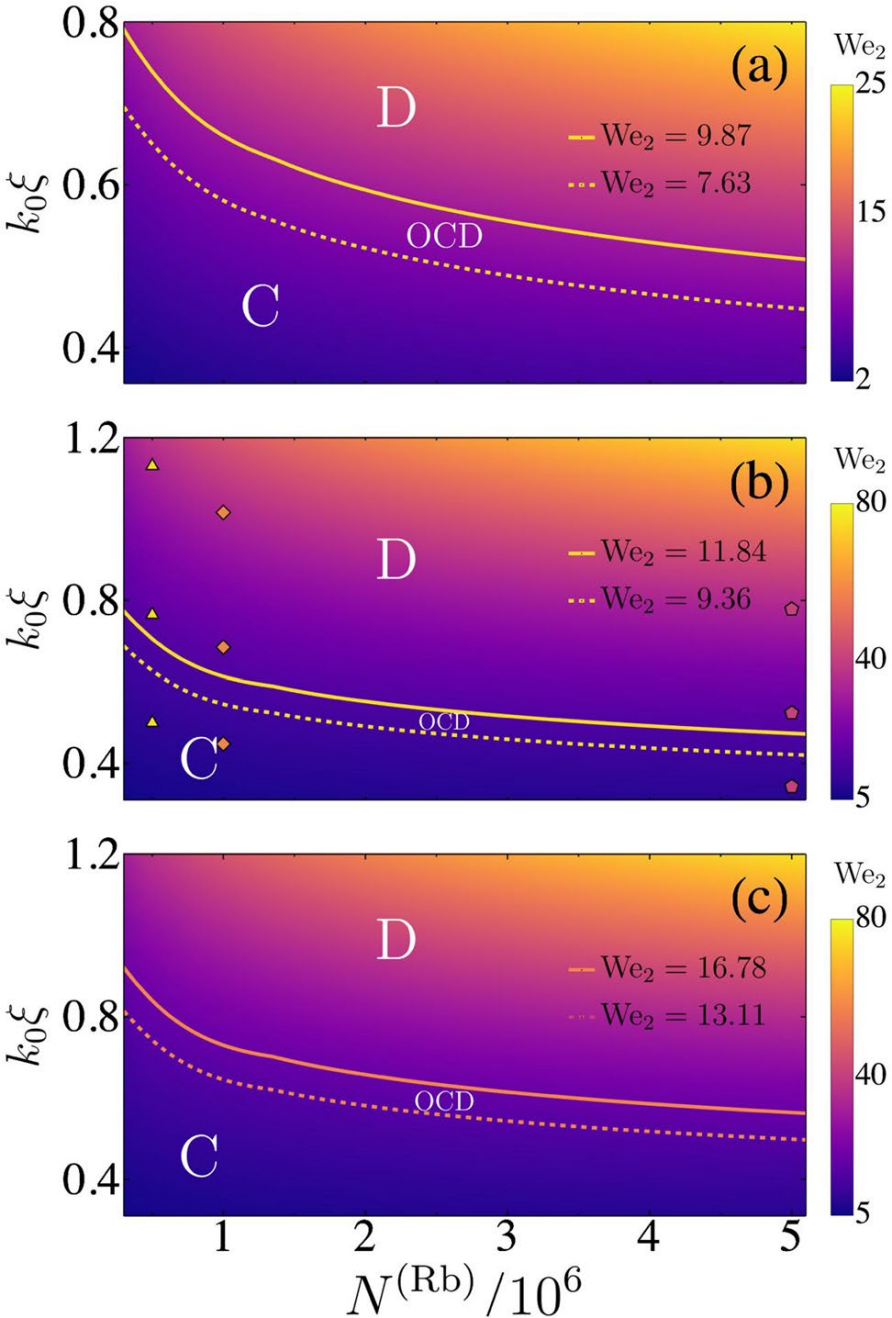
Quadrupole Weber number $\mathrm {We}_2$ as a function of the number of atoms of a given species is illustrated for (**a**) homunuclear and (**b**) hetero-nuclear droplets (**c**) hetero-nucleat droplets including atom losses. In the first case *N* corresponds to half the total number of atoms in the droplet and in case (**b**), (**c**) to $N^{(Rb)}$ in an Rb and K admixture. The outcome of a frontal collision will give rise to the coalescence of the droplets for $\mathrm {We}_2$ below the lower line (C region). The disintegration of the originally droplet formed during the collision is observed for $\mathrm {We}_2$ above the higher line (D region). For values of $\mathrm {We}_2$ between these lines involve a long transient stage where the coalesced droplet oscillates strongly before its disintegration (OCD region). The dots in (**b**) correspond to the values of $k_0\xi$ and $N^{(Rb)}$ illustrated in previous figures.

The Weber number given by Eq. (35) must be evaluated according to the expression of the surface tension adequate for the regime at which the initial droplets are prepared: in the incompressible regime *ansatz* 1 is used while in the compressible regime *ansatz 2* is the correct one. The droplets dynamics seen in the collisions tend to be mainly quadrupolar as can be infered by comparing Fig. (1) to Figs. (3–5), so that the quadrupolar Weber number is proposed as a figure of merit. In the illustrated simulations, $N^{Rb}$ is compatible with excitations described by *ansatz* 1, and the Weber number describes the qualitative outcomes. This assertion is also supported by the numerical calculation of the multipole moment of the quantum liquid after a frontal collision. Figure 6, illustrates the Weber number $We_{\ell =2}$ as a function of the number of atoms and the wave vector $k_0$. When three-body losses are included, the Weber number is still a good parameter to delimit the immediate breakdown of a droplet after the collision. However the transient regime is linked to a different band of $\mathrm {We}_2$ values. A rich variety of transient configurations that culminate in one of the three above results are found. Their observation depends highly on the inclusion of three-body atom losses. In the ideal case $K_{\alpha \beta \gamma } = 0$, frequently after the break into two droplets like that described in (2), the dipole like oscillation of each individual droplet is faster than their relative translation and afterwards coalescence occurs-described by (1), the final single droplet remains oscillating mainly in a quadrupole way. The transient regime usually takes place for quadrupole Weber numbers between two given values $\mathrm {We}_2^{min}$ and $\mathrm {We}_2^{max}$ whose values are exemplified in Fig. 6 for collisions between the droplets. For $\mathrm {We}_2 < \mathrm {We}_2^{min}$ the scenario described by (1) takes place without a transient stage. For $\mathrm {We}_2 > \mathrm {We}_2^{max}$ the scenario described by either (2) or (3) takes place without a transient stage. Figure 6 is supported by extensive numerical simulations carried out for the values of the parameters $k_0\xi$ and *N* reported in it.

When three-body losses are included, the observation of the results of the collision is strongly dependent on the initial conditions. For a given $\vec{k}_0$, the parameter $\vec{d}_0$ should be chosen so that atom losses by three-body scattering are small-less than $2\%$ before the collision takes place- and, simultaneously, the droplets overlap at $t = 0$ is avoided. The mean life time of the hetero-nuclear droplets is in the range of tens of milliseconds as expected from calculations in “Atom losses” section, and confirmed by experimental evidence^6^. So that, the long time evolutions like those described in the ideal case are difficult to observe for low $k_0$ values. Nevertheless, in spite of the short times involved, the regimes at which the scenarios (1)–(3) occur were observed in the simulations. An interesting effect due to the atomic gas that surrounds each one of the droplets is also predicted: the surrounding evaporated gas created nearby a given droplet interact with the atoms from the other droplet through contact terms. This results in a reconfiguration of a merged quantum droplet. The effect is similar to that exemplified by Fig. 1c,d. Taking into account three-body losses, the Weber number is still a good parameter to delimit the immediate breakdown of a coalesced droplet after the collision.

## Discussion

In this work we have studied theoretically the static and dynamical properties of quantum droplets constituted by binary mixtures of homo-nuclear and hetero-nuclear ultracold atoms. One of the more interesting features of quantum droplets corresponds to self-trapping. Our calculations show that the effective equations as those built as extensions to the Gross–Pitaevskii formalism provide robust interpretations of the droplets behavior both in the compressible and the incompressible regime. Our calculations confirm the slight differences between the expectations derived from Monte Carlo studies and the LHY-EGPE. They manifest not only on the minimum number of atoms required for self-trapping, but also on the radius $R_0$ and thickness of the surface *dR* of the droplets; their dependence on the scattering lengths is discussed at depth in Ref.^16,17^. We have also shown that the analytical model Eq. (28) is a reasonable representation of the density of atoms $\rho _0^{(\alpha )}$.

We found that in the incompressible and compressible regimes the elementary Bogolubov excitations, Eqs. (15–17), are better described in a variational basis by Eqs. (18) and (19) respectively. We provide an analytic expresision for these excitations for any given multipole moment $\ell$. The spherical symmetry of the droplets is directly incorporated in the selection of the basis. The corresponding expressions for the surface tension reflect such a symmetry, and exhibit a different behavior on the multipole order. For *ansatz 1*, the $\ell$-dependent coefficient that accompanies the expression of the energy of the excitations coincides with that expected for classical liquids and that of the liquid drop model of nuclei in the limit of $N\rightarrow \infty$ nucleons. Explicit calculations of the excitation energies show that this *ansatz* is the more adequate one to describe the incompressible regime of the quantum droplets. The excitation modes and the surface tension derived from *ansatz 2* are more adequate for the quantum fluid in the compressible regime. Differences in the predicted shape of the droplets between the effective range formulation and the EGPE manifest also on slight differences in the excitation energies of the Bogolubov modes.

Self-evaporation is a particularly interesting phenomenon predicted for quantum droplets that is not easily observed due to its competition with atom losses arising from three-body scattering. We have identified two other relevant mechanisms that accelerate the disintegration of the quantum droplet: loss of the adequate proportion of each atomic species and differences in spatial configuration of each species.

In spite of the difficulties to experimentally study the dynamics of quantum droplets, we have theoretically shown that if the droplets are excited via frontal binary collisions it is still possible to observe a non trivial dynamics. The Weber number resulting from the surface tension expressions introduced in our study exhibit a simple dependence on the radii of the droplets $R_0$ and the relative droplets momentum $\hbar k_0$ which are measured in terms of the surface width *dR*, Eqs. (36, 37). These make explicit the surface character of the emerging excitations. We have also shown that the Weber number evaluated with the adequate *ansatz* define different regimes of excitation that include conditions for (1) the formation of pairs of quantum droplets excited as oscillating dipoles that detach from each other leaving the impact region, (2) the formation of a single droplet (mainly in a quadrupole excitation mode) that merges from two elementary droplets; (3) if three-body losses are included, space regions are created where the lost atoms interact via de contact terms and lead to a reconfiguration of otherwise disjoint droplets (this effect is similar to that exemplified by Fig. SM 3 c-d of the Supplementary Material but it is enhanced by the evaporated atoms). Contrary to analysis that evaluate the surface tension of the planar interface described by local energy functionals and later on incorporate curvature through Tolman like terms^33^, no extra parameters are required within our formalism to identify the different collision regimes.

## Conclusions

The Bogolubov functions introduced in this work and selected via variational criteria, give a reliable representation of the low energy excitations of quantum droplets constituted by binary mixtures with a finite number of homo- and hetero-nuclear atoms. To our knowledge, the dynamical definition of the surface tension of a quantum droplet that we introduce via the Rayleigh interpretation (25) has not been reported yet in the literature. Our *ansatze* give the energy estimation of a surface excitation up to a multipole order $\ell$.

As the results of this work do not depend on the strength of the interactions, albeit the ground state of the droplets, these could be extrapolated to many different droplets with similar geometries. The basis of our work could be applied to different systems such as dipolar droplets, droplets with impurities, or the novel mixed bubble phase^23^.

We have focused our study to low energy surface excitations that can be induced by collisions between ground state droplets. Other interesting excitation modes have been predicted, in particular, vortical quantum droplets that could provide a three-dimensional realization of 3D topological solitons in nonlinear media^35^. In this case, the bulk excitation yield self-trapped vortex tori. It would result very interesting to complement the careful numerical study performed in^35^ by a variational study of these high energy excitations.

Our variational expressions are useful to describe the qualitative and quantitative expectations of frontal binary collisions of the droplets as a function of the most relevant parameters, such as number of atoms, interaction strengths, translation kinetic energy. This panorama is valid even in the presence of atom losses where the Weber number- here defined and calculated—is a good figure of merit to discriminate the expected outcomes of such collisions. Two interesting effects concerning atom losses are predicted, the first corresponds to the saturation of the exponent that describes the time dependence of atom losses via three body scattering (details in SMC). The second effect arises in atom collisions when the surrounding evaporated gas created nearby a given droplet interact with the atoms from the other droplet through contact terms, resulting in a reconfiguration of a merged quantum droplet.

We hope that these theoretical analyses encourages the research of quantum droplets in both the compressible and the incompressible regimes, the latter fairly unexplored due to the experimental difficulties related to its generation and characterization. These theoretical and experimental studies would pave the way to further studies of novel excited states induced in ultracold atoms, and their analog to other quantum matter systems.

## Supplementary Information

Supplementary Information.

## Data Availability

The datasets used and/or analysed during the current study available from the corresponding author on reasonable request.
