# Supplementary material for: Weber number and the outcome of binary collisions between quantum droplets
Source: Sci Rep. 2022 Nov 2;12:18467. doi: 10.1038/s41598-022-22904-8 (PMC9630383; doi:10.1038/s41598-022-22904-8)
Supplement: Supplementary file 1 — Supplementary Information. [file 41598_2022_22904_MOESM1_ESM.pdf]

Supplementary material to
Weber number and the outcome of binary collisions between quantum droplets

J. E. Alba-Arroyo,[1] S. F. Caballero-Benitez,[1] and R. Jáuregui*[1]

[1]*Departamento de Física Cuántica y Fotónica*
Instituto de Física, Universidad Nacional Autónoma de México
Cd. de México C.P. 04510, México
**rocio@fisica.unam.mx*

(Dated: October 26, 2022)

SMA. GROUND STATE DROPPLETS: NUMERICAL SIMULATIONS RESULTS.

We have evaluated numerically the ground state for binary mixtures of atomic Bose gases for a finite number of atoms, ranging from $10^4 - 10^7$ within the conditions on the interaction strengths that lead to quantum droplets. For homo– and hetero–nuclear mixtures, we worked out the EGPE scheme as given by Eq. (38). The effective formalism that considers finite-range effects as developed in Refs. [15,16], was also worked out for the homo-nuclear case.

For a homo–nuclear mixture of $N^{(a)} = N^{(b)} = N/2$ ^{39}K atoms, we considered $\mathrm{a}_{aa} = \mathrm{a}_{bb} = 48.57\mathrm{a}_0$, $\mathrm{a}_{ab} = -51.36\mathrm{a}_0$. $N_c^{(a)} \approx 18.65 \times n_0^{(a)}\xi^3 \approx 37210$ for EGPE. For the hetero–nuclear case, ^{41}K and ^{87}Rb atoms we considered, $\mathrm{a}_{KK} = 62.0\mathrm{a}_0$, $\mathrm{a}_{RbRb} = 100.4\mathrm{a}_0$, $\mathrm{a}_{KRb} = -82.0\mathrm{a}_0$, and $N^{(Rb)}/N^{(K)} \sim 1.15$. The results of our calculations throw $N_c^{(K)} = 19.58 \times n_0^{(K)}\xi^{(a)3} = 11067$. Note that the difference between the predictions of the LHY approach and the density Monte Carlo formalisms are expected to increase as $|\mathrm{a}_{KRb}|$ increases [17]. The range $-85\mathrm{a}_0 < \mathrm{a}_{KRb} < -77\mathrm{a}_0$ minimizes discrepancies. The ideal saturation densities are $n_0^{(K)} = 3.745 \cdot 10^{20}\mathrm{m}^{-3}$ and $n_0^{(Rb)} = 4.287 \cdot 10^{20}\mathrm{m}^{-3}$.

The general properties of the ground state of the EGPE in the droplet regime for homo-nuclear mixtures have been predicted by Petrov, they are compatible with a Boltzmann function shape as an approximate mathematical model of the spherical density profile,

$$\rho_B(R;N) = \mathcal{B}_1/(1 + \exp(R - R_0)/dR)).$$

The parameters R_0 and dR are then interpreted as the radius of the droplet and its surface thickness respectively. The density at $R = 0$,

$$\rho_B(0;N) = \mathcal{B}_1/(1 + \exp(-R_0/dR))$$

is required to fix the $\mathcal{B}_1$ parameter.

For $N^{(\alpha)} > N_c^{(\alpha)}$, the droplet radius fitting with a Boltzmann distribution was found to satisfy

$$R_0(N^{(\alpha)}) = (\mathfrak{r}_0^{(\alpha)} + \mathfrak{u}^{(\alpha)}\big(3N^{(\alpha)}/4\pi n_0^{(\alpha)}\xi^{(\alpha)3}\big)^{1/3})\xi^{(\alpha)},$$

while the density at $R = 0$ satisfies

$$\rho_B(0;N^{(\alpha)}) = [\rho_\infty^{(\alpha)} + \delta\rho_N^{(\alpha)}\exp(-b\,N^{(\alpha)})]n_0^{(\alpha)}, \quad N^{(\alpha)} \geq 3N_c^{(\alpha)}.$$

For the homo-nuclear case, the thickness of the surface results almost independent on $N^{(\alpha)}$ for $3N_c^{(\alpha)} < N^{(\alpha)} \leq 10^7$ and given by $dR = dR_0\xi$ with dR_0 given in Table I.

For the hetero–nuclear mixture, if $N_c^{(\alpha)} < N^{(\alpha)} < 3N_c^{(\alpha)}$, dR decreases as $N^{(\alpha)}$ increases, being always in the interval $dR \in [0.50\xi, 0.61\xi]$. The density at $R = 0$, $\rho_\alpha(0;N^{(\alpha)})$, is a non monotonic function of $N^{(\alpha)}$. Besides, for a given droplet, both species share the same radius and these densities satisfy $\rho_b(0,\infty)/\rho_a(0,\infty) = \sqrt{g_{aa}/g_{bb}}$. We found from the numerical calculations that,

$$\rho_\alpha(0;x^{(\alpha)}) = A_2^{(\alpha)} + \frac{A_1^{(\alpha)} - A_2^{(\alpha)} + g_0(x^{(\alpha)})}{1 + g_1(x^{(\alpha)})}, \quad g_n(x^{(\alpha)}) = \exp\left(\left(x^{(\alpha)} - x_n^{(\alpha)}\right)/dx_n^{(\alpha)}\right), \quad x^{(\alpha)} = (N^{(\alpha)} - N_c^{(\alpha)})^{1/3}.$$

This expression quantifies the characteristic steep exponential growth of $\rho_\alpha(0;x)$ for small x values, followed by a soft decrease after the maximum saturation density is achieved. The latter occurs at $(N^{(Rb)} - N_c^{(Rb)})^{1/3} \approx 42.5$, equivalent to 89435$\approx 7N_c^{(Rb)}$ Rb atoms. For a smaller $N^{(Rb)}$, $R_0 \sim dR$ and the interpretation of dR as an effective width of

the droplet surface is not evident. Therefore, the self–confinement region with a compressible fluid corresponds to $N_c^{(\alpha)} \leq N^{(\alpha)} \leq 7N_c^{(\alpha)}$. For larger $N^{(\alpha)}$ the fluid is approximately incompressible.

The predictions on the properties of the ground state that were mentioned in Ref. [2] for the saturation density, and the general behaviour of R_0 and dR within EGPE, have been numerically tested. The fitting parameters of the ground states of both homo-nuclear and hetero–nuclear are depicted in Table I.

The numerical density profiles found in the calculations fit the Boltzmann density with a reliability always above 0.998 for droplets with $N > N_c$ and even higher for $N > 3N_c$.

Homo–nuclear						
	$\mathfrak{r}_o$	$\mathfrak{u}$	ρ_∞	b	$\delta\rho_N$	dR_0
EGPE	-0.54± 0.01	1.051± 0.004	0.95± 0.17	1/3	0.24± 0.38	0.53± 0.01
Finite range	-0.54± 0.03	1.069±0.006	0.907± 0.005	1/4	0.34± 0.26	0.56± 0.02
Hetero–nuclear						
^{41}K	-0.47±0.02	0.975±0.002	1.032±0.002	1/3	-	0.52±0.01
^{87}Rb	-0.48± 0.02	0.973± 0.002	1.177±0.003	1/3	-	0.52±0.01
$\rho_\alpha(0; x^{(\alpha)})$ Atom	$A_1^{(\alpha)}$	$A_2^{(\alpha)}$	$dx_0^{(\alpha)}$	$dx_1^{(\alpha)}$	$x_0^{(\alpha)}$	$x_1^{(\alpha)}$
^{41} K	1.032 ± 0.002	0.475 ± 0.004	-55.9 ± 0.5	-6.36 ± 0.11	-72.7 ± 1.1	16.59 ± 0.14
^{87} Rb	1.177 ± 0.003	0.450 ± 0.005	-54.6± 0.6	-8.39 ± 0.24	-52.3 ± 1.2	14.3 ± 0.6

TABLE SM I: Parameters of the ground state quantum droplets.

SMB. SELF-EVAPORATION: NUMERICAL SIMULATIONS RESULTS.

Self–evaporation arises whenever the number of atoms in the droplet is such that its excitation energy ϵ lies in the continuum, i.e., $\epsilon > -\mu$. Previous studies [2,16,26] as well as the estimatives of those energies illustrated in Fig. 2, show that this phenomenon occurs preferably in the compressible regime.

The evolution of self-evaporation in homo–nuclear mixtures is illustrated in Fig. 1a. The excitation functions $u_q^{(\alpha)}$ and $v_q^{(\alpha)}$ are assumed as given by the *ansatz* 2 which is variationaly adequate for $N^{(\alpha)}(0) = 100000$. This number of atoms produces stable ground states for both EGPE and MC. *Ansatz* 1 is used for $N^{(\alpha)}(0) = 500000$ which it is also shown in Fig. 1a. It can be observed that the self evaporation rate is not uniform in time and an asymptotic state is reached. In general, near N_c the excitation energy gives rise to enough atom losses to dissociate the droplet. In the incompressible regime, excitations occur in a more defined surface region and the self-evaporation gives rise to lower particle losses. For $N^{(\alpha)}(0) = 100000$, the quantum dropplet reaches a stable state with $N^{(a)}(t \to \infty) \sim 62500$. Our numerical simulations show that this state corresponds to the ground state for that atom number: the excitation energy has been released by atoms losses with a self-confinement final state. For greater values of $N^{(a)}$ the atom loss rate suppresses, until a state is reached where excitations prevail without significant self-evaporation.

For the hetero–nuclear mixtures, illustrated in Fig. 1b, the self-evaporation curve exhibits richer time and atom number structures. First, let us focus on values of $N^{(a)}(t = 0)$ for which $\mu_{\mathrm{K}} < \mu_{\mathrm{Rb}}$. Being in the compressible regime, natural excitations are better described by *ansatz* 2. At short times, more K than Rb atoms evaporate with a slight difference. Afterwards, the rates become even more similar reaching complete evaporation. Out of the compressible regime, with natural excitation modes described by *ansatz* 1, both ^{87}Rb and ^{41}K evaporation rates diminish, with an asymptotic droplet ground state of fewer atoms. At transient times, the relative atomic release rate oscillates. This seems to follow from single atom losses and commensurability of $N^{(a)}/N^{(b)}$. For larger $N^{(a)}(0)$, self-evaporation is suppressed, the normal mode excitation is allowed, and the atomic droplets behave as expected from the ideal evolution, see Section IV.

Numerical simulations show that in the early stages, the evolution of the quantum droplets in the compressible regime is similar to that expected in the ideal case, that is, without self evaporation. In Figs. 2-3 we illustrate those ideal profiles for two particularly interesting cases. Figure 2 corresponds to a droplet at the onset of self-trapping and Figure 3 corresponds to $N^{(\alpha)} \sim 3.5N_c^{(\alpha)}$ at which the excitation energy is similar to the negative of the chemical potential. Notice that for $N \sim N_c^{(\alpha)}$ the deformation of the droplet with respect to the ground state as a function of time is less pronounced for A2 than for A1. This is consistent with a compressible character of the quantum fluid for this number of atoms. For $N^{(\alpha)} \sim 3.5N_c^{(\alpha)}$ the comparative evolution of the droplets is similar at several times. Since

self-evaporation occurs in a milisecond scale that allows that 82% of the atomic cloud illustrated in Fig. 3 remains in such a sphere for times as long as 100ms. In the case of the cloud illustrated in Fig. 2 this time reduces to 15 ms. Taking into account the values of the excitation energies explictly given in the captions of the figures, this would permit to observe about one complete oscillation for the lowest number of atoms that yield the self-trapping condition, and about 10 oscillations for the droplet with $N^{(\alpha)} \sim 3.5 N_c^{(\alpha)}$.

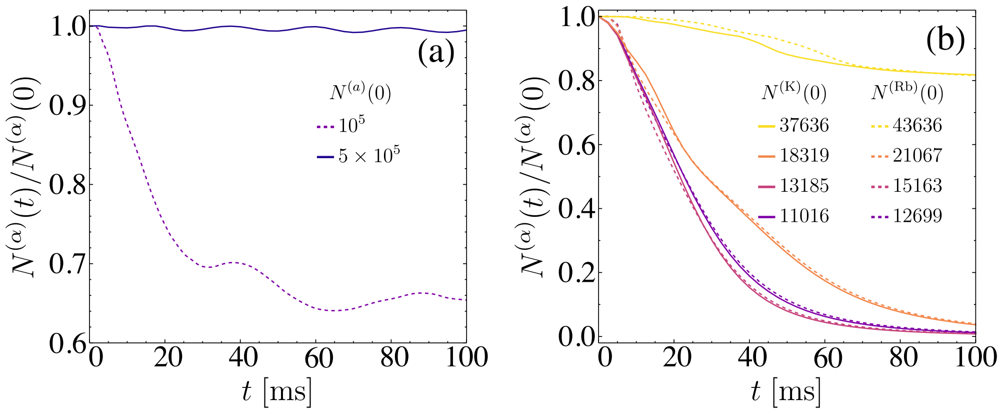

Fig. SM 1: Number of atoms evaluated from EGPE without losses expected from three–body scattering that remain within a sphere with the minimum radius necessary to include the complete atom cloud under an ideal quadrupole excitation (a) for a homo–nuclear mixture of ^{39}K atoms, (b) for a hetero–nuclear mixture of ^{41}K and ^{87}Rb atoms.

SMC. THREE-BODY LOSES: NUMERICAL SIMULATIONS RESULTS.

Three-body scattering effects are studied by solving the LHY-EGPE with the addition of imaginary terms $iK_{\alpha\beta\gamma}\Psi_\alpha\Psi^*_\beta\Psi_\gamma$ that emulate atom losses. We performed numerical simulations of the evolution of the quantum droplet consistent with experiments. A confining potential was added, avoiding the atom losses from the trap at short times. Later, this potential is turned off and the evolution is numerically followed. For homo–nuclear mixtures of ^{39}K, the biggest $K_{\alpha\beta\gamma}$ values correspond to collisions between atoms in the same hyperfine state [4,5,11], $K_{111} = 6 \times 10^{-41} m^6/s$ and $K_{222} = 5.4 \times 10^{-39} m^6/s$. For hetero–nuclear mixtures of ^{41}K - ^{87}Rb the three–body losses are smaller [6]: the dominant channel of losses correspond to K-Rb-Rb scattering, $K_{KRbRb} = 7 \times 10^{-41} m^6/s$. The time evolution of the atom losses was estimated with the initial state as the ground state of a droplet obtained from the EGPE. To monitor the losses, the number of atoms that remain within a sphere of radius $R_0 + dR$ was used, following data in Section SMA. An exponential fit with a parameter $\lambda^{(a)}$ that depends on the atomic species a and the initial number of atoms N_a were evaluated. We considered $1 \times 10^4 \leq N^{(Rb)}(0) \leq 1.5 \times 10^6$, and as usual $N^{(K)}(0)/N^{(Rb)}(0) = \sqrt{g_{RbRb}/g_{KK}}$. It could be observed that as $N^{(Rb)}(0)$ increases, the exponential fit parameter $\lambda^{(\alpha)}$ also increases but with an always diminished rate. A reliable logistic fit $\lambda^{(\alpha)} := \lambda^{(\alpha)}(N^{(\alpha)})$ in units of ms^{-1} was made. The results for the conditions described in Subsection IV C yielded: $\lambda^\alpha = l_1 - l_2/(1 + N^{(\alpha)}(0)/l_3)^{l_4}$, numerical results are depicted in Table II. The decaying exponents saturate as $N^{(\alpha)}(0)$, $\alpha =$K, Rb increase. Three body losses for hetero-nuclear mixtures were studied in Ref. [26]. Those calculations do not involve enough initial number of atoms to make evident the saturation effects here found. Nevertheless, losses induced by three–body scattering still involve greater rates than self-evaporation. This follows from ^{41}K - ^{87}Rb proportions breaking the condition for self-trapping.

Atom	l_1	l_2	l_3	l_4
^{41} K	0.0264 ± 0.0003	0.0475 ± 0.0025	16600 ± 2000	0.47 ± 0.02
^{87} Rb	0.0118± 0.0001	0.0173 ± 0.0014	22800± 4100	0.73 ± 0.04

TABLE SM II: Parameters of the decaying exponent $\lambda^{(\alpha)}$.

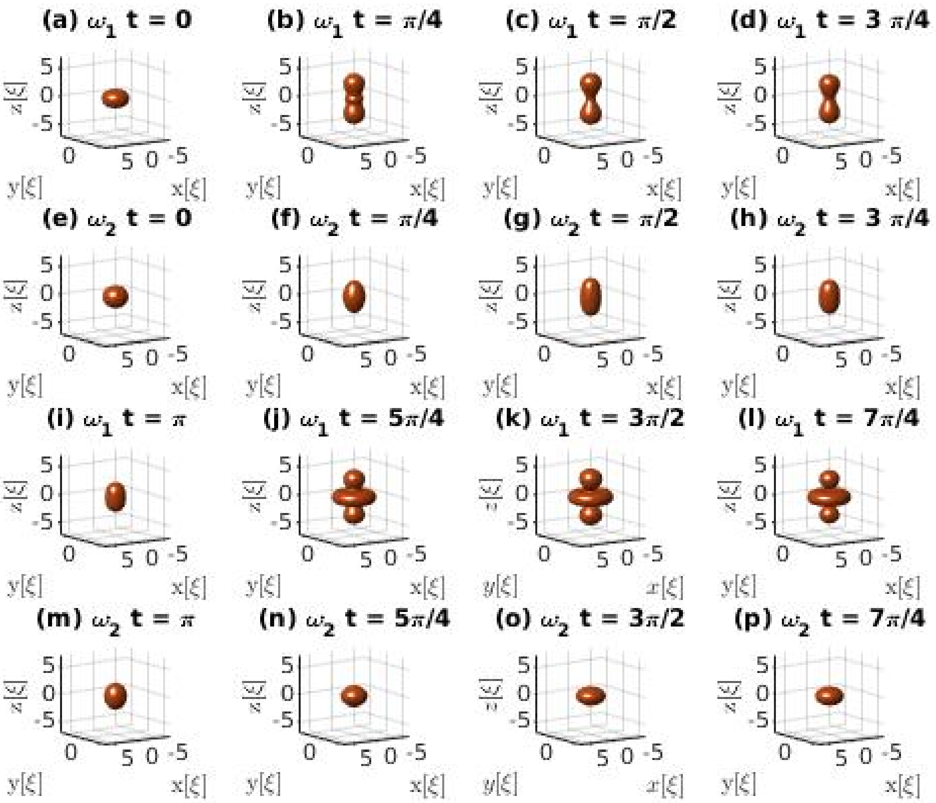

Fig. SM 2: Comparative illustrations of the ideal evolution of the density of a quantum droplet formed by $N^{(Rb)} = 12699$ and $N^{(K)} = 11067$ atoms considering the excited quadrupole state with $m_\ell = 0$ generated from the ground state droplet. *Ansatz* 1 corresponds to rows (a-d) and (i-l)) and *ansatz* 2 to rows (e-h) and (m-p). In this case $\omega_1 = 0.56\tau^{-1} \sim 0.47\text{ms}^{-1}$ and $\omega_2 = 0.36\tau^{-1} \sim 0.30\text{ms}^{-1}$. The 3D graphs where generated by plotting isodensity surfaces at fifteen percent of the maximum density at each time.

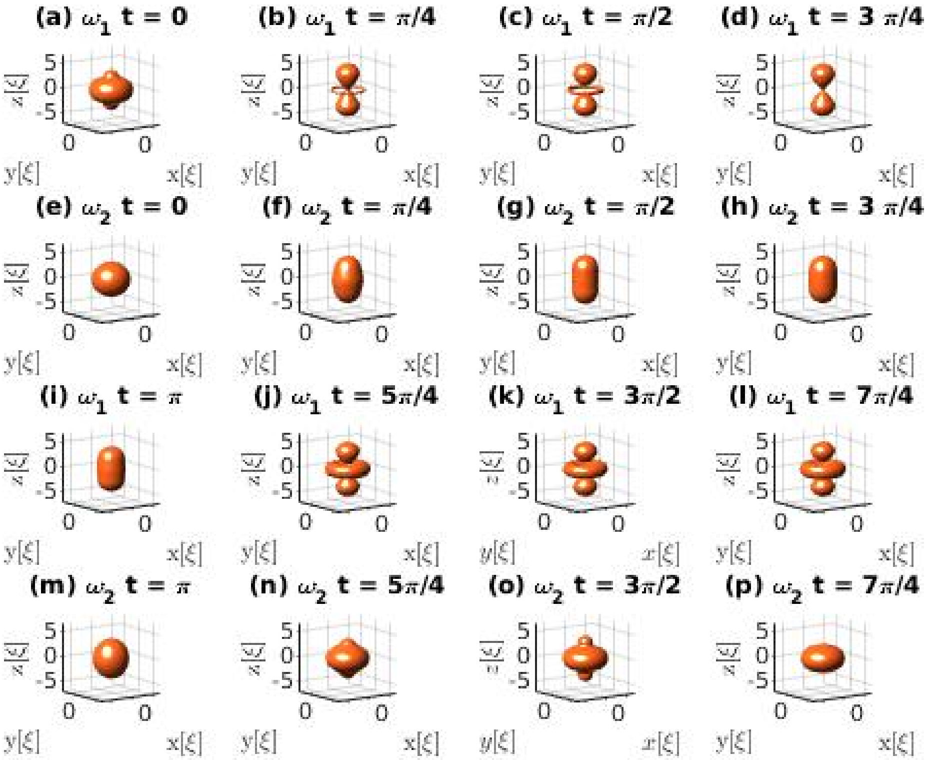

Fig. SM 3: Comparative illustrations of the ideal evolution of the density of a quantum droplet formed by $N^{(Rb)} =$ 43636 and $N^{(K)}$=38119 atoms corresponding to the excited quadrupole state with $m_\ell = 0$ generated from the ground state droplet. *Ansatz* 1 corresponds to rows (a-d) and (i-l) and *ansatz* 2 to rows (e-h) and (m-p). In this case $\omega_1 = 0.44\tau^{-1} \sim 0.37\text{ms}^{-1}$ and $\omega_2 = 0.42\tau^{-1} \sim 0.35\text{ms}^{-1}$. The 3D graphs where generated by plotting isodensity surfaces at fifteen percent of the maximum density at each time.
